# The IL-1 cytokine family and its role in inflammation and fibrosis in the lung

**DOI:** 10.1007/s00281-016-0559-z

**Published:** 2016-03-21

**Authors:** L. A. Borthwick

**Affiliations:** Fibrosis Research Group, Institute of Cellular Medicine, Newcastle University, 4th Floor, William Leech Building, Newcastle upon Tyne, NE2 4HH UK

**Keywords:** IL-1, IL-18, IL-33, Fibroblast, Inflammation, Fibrosis, Lung

## Abstract

The IL-1 cytokine family comprises 11 members (7 ligands with agonist activity, 3 receptor antagonists and 1 anti-inflammatory cytokine) and is recognised as a key mediator of inflammation and fibrosis in multiple tissues including the lung. IL-1 targeted therapies have been successfully employed to treat a range of inflammatory conditions such as rheumatoid arthritis and gouty arthritis. This review will introduce the members of the IL-1 cytokine family, briefly discuss the cellular origins and cellular targets and provide an overview of the role of these molecules in inflammation and fibrosis in the lung.

## Introduction

Progressive loss of organ function due to fibrosis contributes significantly to the ever-growing burden of chronic disease in the world. In addition, because progression in these diseases occurs over several years, they account for enormous morbidity within society, major health resource utilisation and loss of working days and tax revenues. For example, the total cost of respiratory disease in the 28 countries of the EU alone amounts to more than €380 billion annually (European Lung White Book, Chapter 2). Improving our understanding of the processes that fail to orchestrate physiological repair in the injured organ and instead cause pathological repair as fibrosis will aid the identification of new therapeutic approaches that can impact in this area.

Fibrosis causes excessive collagen and extra-cellular matrix deposition in an organ or tissue as part of an attempted reparative process following injury. It may represent an aberrant response to a single large injury but more commonly is a response to a persistent or repetitive injury. The association of fibrosis with many chronic inflammatory conditions suggests that the beneficial reparative processes that restore tissue homeostasis in physiological healing continue unchecked and instead result in pathological damage and loss of organ function. Chronic lung disease is a very common cause of morbidity and mortality across Europe, with diseases such as idiopathic pulmonary fibrosis (IPF) accounting for >2500 deaths a year and chronic obstructive pulmonary disease (COPD) causing over 30,000 deaths/year in the UK with a rising incidence (predicted to become the third common cause of death by 2020) [1, 2]. Fibrosis causes significant dysfunction of the lung by remodelling the small airways in COPD contributing to airflow limitation [3] and by remodelling the parenchyma in IPF severely impairing gas exchange [4]. Both conditions are associated with significant inflammation, yet the mechanisms linking inflammation to fibrosis in the lung are poorly understood.

**Fig. 1:**
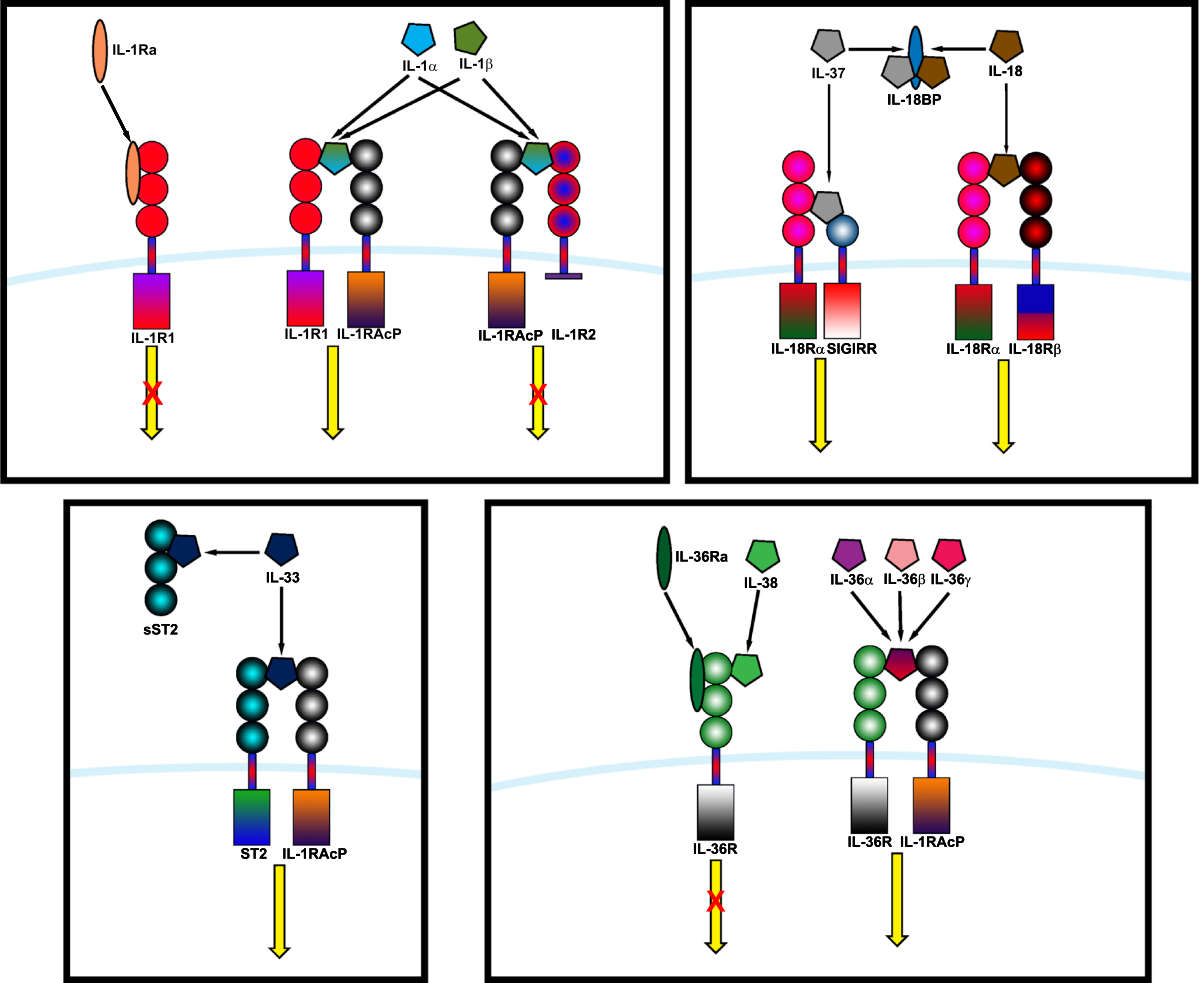
IL-1 family cytokines and their known receptors. The IL-1 cytokine family consists of seven agonists (IL-1α, IL-1β, IL-18, IL-33, IL-36α, IL-36β and IL-36γ), three antagonists (IL-1Ra, IL-36Ra and IL-38) and one anti-inflammatory cytokine (IL-37). The known interactions of the IL-1 cytokines with the IL-1 receptor family are summarised above

The IL-1 family consists of 11 members, 7 of which have been demonstrated to have broad pro-inflammatory activity (IL-1α, IL-1β, IL-18, IL-33, IL-36α, IL-36β and IL-36γ) while the remaining 4 have antagonistic (IL-1Ra, IL-36Ra, IL-38) or anti-inflammatory (IL-37) properties. The IL-1 receptor family comprises 10 members, namely, IL-1R1, IL-1R2, IL-1R accessory protein (IL-1RAcP), IL-18Rα, IL-18Rβ, ST2 (IL-33R), IL-36R (previously IL-1Rrp2), single Ig IL-1R-related molecule (SIGIRR or TIR8), three Ig domain-containing IL-1R related-2 (TIGIRR-2 or IL-1RAPLI) and TIGIRR-1 (IL-1RAPL2). A simplified nomenclature for IL-1 receptor family members (IL-1R1–IL-1R10) was recently proposed by Garlanda et al. in Immunity [5]. The known interactions between the IL-1 family members and the IL-1 receptor family are summarised in Fig. 1.

In this review, I will introduce the members of the IL-1 cytokine family, briefly discuss the cellular origins and cellular targets and provide an overview of the role of these molecules in inflammation and fibrosis in the lung. Where appropriate, readers will be directed to more substantial reviews that focus on specific topics in more detail. Additionally, although the IL-1 cytokine family has been shown to be important in inflammation and fibrosis in numerous organs, this review will primarily focus on the role of the IL-1 cytokine family in the lung.

### IL-1 subfamily

#### IL-1 and IL-1Ra

In the 1940s, during the search for the endogenous mediator of fever, researchers identified a small soluble candidate protein released by activated white blood cells that was termed leukocytic pyrogen [6]. In 1977, leukocytic pyrogen was purified [7] and was demonstrated to be identical to lymphocyte activation factor [8], a factor released from stimulated mononuclear cells that augmented lymphocyte proliferation [9]. The term interleukin was introduced in 1979 to consolidate the nomenclature for factors with the ability to act as communication signals between different populations of leukocytes, and the term IL-1 was assigned to leukocytic pyrogen/lymphocyte activation factor [10]. Several years’ later, two sequences for IL-1 cDNA were cloned in mice and humans and were subsequently named IL-1α and IL-1β [11, 12]. The genes for IL-1α and IL-1β are located adjacent to one another on the long arm of chromosome 2 and encode proteins of 271aa and 268aa (∼31 kDa) respectively [13]. Although both proteins bind to the same receptor (IL-1R1), triggering the recruitment of IL-1RAcP to form a signal transducing complex [14], there are several differences in their expression and proteolytic processing that impact on their respective roles in vivo.

IL-1α is a dual-function cytokine, meaning that in addition to functioning as a classical cytokine via cell surface receptor ligation, full-length IL-1α can also directly regulate gene expression. For example, full-length IL-1α contains a nuclear localisation site within its N-terminal amino acid sequence [15] and has been shown to facilitate pro-inflammatory gene transcription independent of IL-1R1 binding [16]. Full-length IL-1α is constitutively expressed in the cytoplasm and nuclei of a range of non-haematopoietic cells such as epithelial cells in a range of tissues including the lung, liver and kidney [17]. Damage to the epithelium of the lung as a result of infection with bacteria or virus [18, 19] or by non-infective insults such as air pollution, oxidative stress, aspiration injury or cigarette smoke results in the release of IL-1α from stressed/necrotic cells into the extracellular space [20–24]. The release of IL-1α from necrotic cells is unique as cells directed to the apoptotic pathway concentrate IL-1α in dense nuclear foci and therefore it is not released along with the cytoplasmic contents during apoptosis [25, 26]. Full-length IL-1α, unlike full-length IL-1β, is biologically active and functions as a damage-associated molecular pattern (DAMP) or an ‘alarmin’ by binding to IL-1R1 and rapidly initiating the production of chemokines and inflammatory cytokines, driving ‘sterile inflammation’ [27, 28]. Therefore, nuclear retention of IL-1α is likely a mechanism to distinguish between apoptotic and necrotic cell death and to limit sterile inflammation in response to programmed cell death. As well as being constitutively expressed in epithelial cells, numerous other cell types have been shown to upregulate and release IL-1α in response to a range of different stimuli including macrophages [29, 30], monocytes [31, 32] and endothelial cells [33] among others [34].

IL-1β is an inducible cytokine and is not generally expressed in healthy cells or tissue; however, full-length IL-1β is rapidly induced in cells by activation of pattern recognition receptors (PRRs) such as TLRs by pathogen products or factors released by damaged cells, leading to intracellular accumulation of the protein [35]. Processing of the full-length precursor to the biologically active mature form of the protein requires caspase-1 which cleaves the N-terminal 116 amino acids from the precursor to generate a mature active cytokine [36]. In the majority of cell types, caspase-1 is maintained in an inactive state and therefore the secretion of active IL-1β is tightly regulated. The inflammasome is a multi-protein intracellular complex that is required for the conversion of pro-caspase-1 to active caspase-1, and has been shown to be required for the cleavage and release of mature IL-1β [37]. However, inflammasome-independent processing of IL-1β has also been demonstrated in caspase-1-deficient mice with neutrophil proteases including elastase, proteinase-3, granzyme A, and cathepsin G able to convert the IL-1β precursor to the active mature protein extracellularly [38, 39]. Pro-IL-1β lacks an N-terminal secretory signal or leader sequence and therefore is not secreted via the classical endoplasmic reticulum−Golgi pathway [40]. Despite over two decades of research, the mechanism by which the active IL-1β protein is secreted from cells remains elusive although several potential mechanisms have been proposed including exocytosis of secretory lysosomes [41, 42], microvesicle shedding from the plasma membrane [43, 44], shedding of multivesicular bodies containing exosomes [45] and direct efflux across hyperpermeable plasma membranes during pyroptotic cell death [46].

Once extracellular, IL-1α and mature IL-1β bind to the IL-1R1 to initiate downstream signalling in a range of cell types [14]. However, additional control on the actions of IL-1α and IL-1β at the level of receptor interactions is provided by IL-1Ra and IL-1R2. The gene for IL-1Ra, like those encoding IL-1α and IL-1β, is located on chromosome 2 in humans, and the similarities in the intron–exon organisation suggests that the IL-1Ra gene originated as a consequence of gene duplication [47, 48]. IL-1Ra binds to IL-1R1 with comparable affinity to IL-1α and IL-1β but fails to induce the conformational change in the receptor that is required for the recruitment of the IL-1RAcP co-receptor and signal transduction [49, 50]. Consequently, IL-1Ra acts as a competitive inhibitor for IL-1R1 and completely inhibits the activity of IL-1α and IL-1β. IL-1R2 is expressed in numerous cell types in a range of tissue in both humans and mice [48]. Also located on chromosome 2, the gene encodes a 68-kDa (compared to the 80-kDa IL-1R1) membrane-bound protein with a truncated (29 amino acids) cytoplasmic tail that is lacking the TIR domain and is therefore incapable of intracellular signalling [51]. IL-1R2 can also be proteolytically cleaved in the proximal extracellular region to generate a soluble receptor (sIL-1R2) [52]. Although IL-1R2 binds to IL-1α, IL-1β and IL-1Ra, the binding affinity for IL-1Ra is ∼100-fold less than for IL-1α and IL-1β [53] and therefore IL-1R2 is likely to further enhance the inhibitory action of IL-1Ra. For example, reducing IL-1R2 expression in keratinocyte by RNA interference increased IL-1-mediated CCL2 and CCL5 mRNA and protein expression confirming the inhibitory effect of IL-1R2 [54].

Recombinant IL-1α and IL-1β has been shown to have potent effects on a range of cell types in vitro, and therefore any unique effects attributed to IL-1α and IL-1β are likely due to differences in the expression and/or release of the biologically active proteins rather than receptor binding or downstream signalling events. For example, work from our group and others has demonstrated that fibroblasts are induced to secrete a range of inflammatory cytokines in response to challenge with both recombinants IL-1α and IL-1β. However, damaged lung epithelial cells release large quantities of biologically active IL-1α, but little to no mature IL-1β, and therefore it is IL-1α that acts as the primary fibroblast stimulatory factor in this setting [55, 56]. In contrast, peritoneal and alveolar macrophages have been demonstrated to regulate fibroblasts through the release of IL-1β, with little to no requirement for IL-1α [57, 58]. Both IL-1α and IL-1β have also been shown to directly stimulate collagen synthesis and proliferation in fibroblasts [59, 60]. However, this function is controversial as there is a large body of literature demonstrating an inhibition of collagen synthesis [61, 62], and therefore further research is required to clarify this contradiction. IL-1β has also been shown to enhance TGF-β1-driven epithelial-to-mesenchymal transition in vitro highlighting another potential mode of action for the cytokine [63, 64]. However, the in vivo relevance of epithelial to mesenchymal transition has been challenged by a number of lineage tracing studies showing that mesenchymal cells in fibrotic tissue do not have an epithelial origin [65, 66], and therefore further research is required to establish the potential in vivo relevance of EMT-derived mesenchymal cells in fibrosis.

Despite the aforementioned differences, both IL-1α and IL-1β have been demonstrated to play important roles in fibrosis in vivo. In 2001, Kolb et al. used adenoviral gene transfer to transiently overexpress IL-1β in rat lungs which led to an acute inflammatory response with severe progressive tissue fibrosis [67]. Subsequently, numerous studies have shown that administration of IL-1β can drive a fibrotic response in the lung and that the degree of fibrosis is of a similar magnitude to that induced by bleomycin challenge [68, 69]. Intratracheal challenge of wild-type mice with human recombinant IL-1α induced neutrophilia, myeloperoxidase activity and inflammation, all of which were abrogated in mice overexpressing IL-1Ra [70]. Similar effects of intratracheal IL-1α challenge were also seen in ventilated preterm lambs [71] and in rats where IL-1α intratracheally causes an acute, neutrophil-dependent, oxidative lung leak that closely resembles human acute lung injury [72]. Furthermore, both IL-1α and IL-1β have been shown to be increased in mice following bleomycin challenge and IL-1α-, IL-1β- and IL-1R1-deficient mice are protected from bleomycin-induced pulmonary fibrosis [55, 68]. Neutralisation of IL-1β has also been shown to attenuate silica-induced inflammation and fibrosis by inhibiting other inflammatory and fibrogenic mediators and modulating the Th1/Th2 balance [73].

Mice overexpressing IL-1Ra have a reduction in the incidence and severity of collagen-induced arthritis whereas mice lacking IL-1Ra have an earlier onset of collagen-induced arthritis [74]. Administration of IL-1Ra by an osmotic minipump in mice exposed to intra-tracheal instillation of bleomycin or silica completely prevented lung damage and the increase in collagen deposition seen in control mice [75]. A similar inhibition of bleomycin-induced lung pathology was seen with Anakinra [68], an IL-1 receptor antagonist used to treat patients with rheumatoid arthritis [76]. Using both IL-1α- and IL-1β-deficient mice and neutralising antibodies, Botelho et al. demonstrated a novel role for IL-1α in murine models of COPD. The authors conclusively demonstrate that neutrophilic inflammation induced by cigarette smoke exposure is IL-1α dependent, but IL-1β and caspase-1 independent. Furthermore, the authors used bone marrow chimeric mice to demonstrate that IL-1R1 expression on non-hematopoietic cells is required to induce inflammation following cigarette smoke exposure [77].

The inflammasome has also been demonstrated to be important in the pathogenesis of lung fibrosis. For example, Gasse et al. demonstrated that mice deficient in apoptosis-associated speck-like protein containing a CARD (ASC), an important component of the inflammasome, have attenuated IL-1β production and are protected from bleomycin-induced lung fibrosis suggesting that one of the mechanisms of action of bleomycin is via the activation of the inflammasome [68]. In a subsequent study, the authors found that uric acid was released into the lung parenchyma following bleomycin challenge and that a reduction in uric acid levels lead to a decrease in IL-1β production, inflammation and fibrosis [78]. The authors postulated that precipitation of uric acid and the resultant formation of monosodium urate crystals could cause membrane damage to cells leading to inflammasome activation and downstream release of IL-1β. Subsequently, the authors demonstrated that administration of exogenous uric acid crystals recapitulates lung inflammation and repair via an inflammasome and IL-1R1-dependent pathway [78].

Asbestos and silica are sensed by the NLRP3 inflammasome leading to the subsequent activation and secretion of active IL-1β [79, 80]. NLRP3-deficient mice demonstrate a reduction in immune cell recruitment to the lungs and a lower inflammatory cytokine burden in a model of asbestosis inhalation [80]. Significantly, both NLRP3- and ASC-deficient mice are protected from silica-induced collagen deposition confirming an important role for the inflammasome in silica-induced lung fibrosis. A recent study by dos Santos et al. employing vimentin-deficient mice has highlighted a novel role for vimentin in Lipopolysaccharides (LPS)-induced caspase-1 activation and IL-1β maturation via interactions with the NLRP3 inflammasome. The authors demonstrate that vimentin-deficient mice are protected from asbestos- and bleomycin-induced lung injury and fibrosis and that vimentin-expressing bone-marrow-derived cells are important for bleomycin-induced activation of the NLRP3 inflammasome and pulmonary fibrosis [81].

The potential therapeutic value of targeting IL-1α and/or IL-1β in a range of lung diseases becomes clear when reviewing the literature.

Development of the chronic inflammatory airway pathology characteristic of COPD is thought to be triggered by inhalation of noxious particles, primarily cigarette smoke which accounts for ∼80–90 % of cases in the USA [82]. Healthy smokers have significantly elevated levels of IL-1β in their bronchoalveolar lavage (BAL) compared to healthy non-smokers, with IL-1β levels correlating in a cigarette-dose-dependent manner [83]. Increased levels of IL-1β in induced sputum from patients with stable COPD have also been reported [84], and in a subsequent study, the number of IL-1α- and IL-1β-positive cells was shown to be increased in biopsy samples taken from GOLD I/II COPD patients compared to non-COPD controls. In addition, levels of IL-1α and IL-1β in sputum and lung tissue of COPD patients are increased [22], while levels of IL-1α and IL-1β correlated during stable disease, at the onset of exacerbation and post-exacerbation [77]. Uric acid, which has been demonstrated to play an important role in lung fibrosis in mice via an inflammasome- and IL-1R1-dependent pathway, has also been reported to be elevated in BAL of healthy smokers compared to healthy non-smokers as well as COPD patients who smoke compared to healthy smokers [85, 86]. Furthermore, serum uric acid has been shown to be associated with increased 30-day mortality and risk for acute exacerbations and hospitalisation in COPD patients [87].

Several studies have presented evidence of activation of the inflammasome in COPD. For example, increased caspase-1 activation was observed in lung samples from smokers and emphysema patients, compared with non-smokers [88]. Inhibition of caspase-1 significantly reduces inflammation in response to challenge with cigarette smoke in animal models [89]. Various molecules that activate the inflammasome have also been shown to be elevated in COPD patients. For example, extracellular ATP activates the NLRP3 inflammasome by engaging the purinergic P2X_7_ receptor [90], and the P2X_7_ receptor is upregulated on alveolar macrophages and blood neutrophils from patients with COPD [91]. In addition, extracellular ATP is elevated in BAL fluid from patients with COPD as compared with normal control subjects and ATP concentrations correlate with an increase in airway infiltration and is associated with a decline in lung function [91, 92]. Together, these data suggest a role for the inflammasome in contributing to the airway inflammation seen in COPD.

IL-1β has also been shown to be elevated in BAL and lung biopsies from IPF patients compared to normal volunteers [69]. Furthermore, in both serum and BAL, a decreased IL-1Ra/IL-1β ratio was found in IPF patients compared to healthy controls, and this imbalance might contribute to a proinflammatory environment in IPF lungs [93]. Autoantibodies against IL-1α were detected in the sera of 11/11 rapidly progressive IPF patients on the 21st hospital day following admission for severe symptoms (compared to 5/11 on the first hospital day) suggesting that extracellular IL-1α may be an undesirable factor in fibrotic lung diseases [94]. Alveolar macrophages isolated from patients with asbestosis, sarcoidosis and IPF have higher levels of IL-1β mRNA and spontaneous IL-1β secretion compared to control subject [95, 96]. In contrast IL-1Ra release from ILD alveolar macrophages was reduced compared to healthy controls, further increasing the IL-1β/IL-1Ra ratio. Importantly, a high IL-1β/IL-1Ra ratio was shown to correlate with BAL cellularity and was associated with advanced or active disease [96]. Similar to COPD, there is data indicating an important role for the inflammasome in the development of IPF. For example, the expression of both NLRP3 and caspase-1 are elevated in unstimulated macrophages isolated from the BAL of IPF patients compared to controls [97]. Additionally, exogenous ATP is elevated in the BAL of stable IPF patients compared to controls and was further elevated in patients with exacerbated IPF [98].

Recently, the US FDA approved the first two drugs proven to slow progression of IPF: pirfenidone (Esbriet by Roche) and nintedanib (Ofev by Boehringer Ingelheim). Nintedanib is a small molecule tyrosine kinase inhibitor while the precise mechanism of action of pirfenidone is incompletely understood. Both nintedanib and pirfenidone have been shown to inhibit lung fibrosis in murine models, and these effects were associated with a reduction in IL-1β levels in lung tissue [99, 100]. This inhibition of IL-1β may help to dampen the profibrotic milieu in the lung, and improving our understanding of whether this reduction in IL-1β is important for the inhibitory action of nintedanib and pirfenidone will be extremely informative.

The primary limitation to survival after lung transplantation is the development of bronchiolitis obliterative syndrome (BOS), a chronic inflammatory process characterised by fibroproliferation, deposition of extracellular matrix and obliteration of the airways. Levels of IL-1Ra were elevated in patients with BOS compared to healthy lung transplant recipients and patients with acute rejection [101]. Furthermore, work in my laboratory has demonstrated that IL-1α and IL-1β concentrations are elevated in BAL samples acquired immediately prior to BOS diagnosis compared to patient-matched BAL samples acquired at other times following transplant or in BAL samples acquired from stable transplant controls [102, 103]. Further investigation of whether these markers contribute to disease pathology or are simply markers of disease progression is required.

#### IL-33 and ST2

Unlike most IL-1 family cytokines which are located on chromosome 2, the gene for IL-33 is found on chromosome 9 (or chromosome 19 in mice) where it encodes a peptide of 270aa corresponding to a full-length protein of ∼30 kDa [104]. The full-length IL-33 protein contains a non-classical nuclear localisation sequence (aa1-75 in human IL-33) and a non-classical homeodomain-like helix-turn-helix DNA binding domain in the same region with a chromatin binding domain (aa40-58 in human IL-33) [105–107]. Consequently, the full-length protein translocates to the nucleus where it has intracrine gene regulatory functions by interacting with and modulating the function of a number of proteins [108]. For example, full-length IL-33 can function as a transcriptional repressor by binding to the N-terminus of the p65 NF-kappaB subunit, inhibiting binding of p65 to its promoter sequence and therefore impairing transactivation of NF-kappaB-regulated genes including IL-1 and TNF [109]. This function is exclusive to the full-length IL-33 protein as the mature IL-33 protein (aa110-266 in human) losses the nuclear localisation and DNA/chromatin binding domains during proteolytic processing.

Although full-length IL-33 is biologically active, cleavage generates mature forms of IL-33 with up to 10-fold more biologically active compared to the full-length protein [110]. It was originally proposed that, like other members of the IL-1 cytokine family, caspase-1 was responsible for cleavage of the full-length protein to active mature forms [104]. However, the predicted cleavage site for caspase-1 is not conserved in IL-33, and further studies have suggested inactivation of IL-33 following maturation by caspase-1 [111]. Indeed cleavage sites in IL-33 more closely resemble those for caspase-3 and caspase-7, proteins activated during apoptosis, and cleavage of full-length IL-33 by caspase-3 and caspase-7 generates biologically inactive breakdown products suggesting that IL-33 is destroyed in apoptotic cells [108, 112, 113]. However, extracellular full-length IL-33 can be matured by calpain, neutrophil elastase and cathepsin G to generate biologically active forms of the protein (aa95-270, aa99-270, aa109-270) with greater activity than the full-length IL-33 [110, 114, 115].

ST2, previously known as an orphan receptor of the IL-1 receptor family, was identified as the IL-33 receptor in 2005 [104]. Binding of IL-33 to ST2 drives a conformational change in ST2 leading to the binding of the IL-33-ST2 dimer to IL-1RAcP and downstream signalling via MyD88 [116, 117]. In addition to membrane-bound ST2, the ST2 gene in humans encodes at least two additional spliced isoforms, sST2 and ST2V. Characterised by the lack of both a transmembrane domain and an intracellular TIR domains, sST2 is released from the cells to act as a soluble decoy receptor by sequestering IL-33 and preventing its binding to membrane-bound ST2 [118]. While ST2 and sST2 are widely expressed throughout tissues, the expression of ST2V is limited primarily to stomach, small intestine and colon [119]. The exact role for ST2V in vivo requires further investigation; however, it can be found integrated into the plasma membrane when transfected into COS7 cells suggesting a possible role in modifying the IL-33-ST2 signalling complex [119].

Although the expression of IL-33 was originally thought to be restricted to constitutive expression in a small number of cell types including endothelial cells [120], it is now clear that IL-33 is much more abundant (fibroblasts, epithelial cell, macrophages, among others) and that its expression can be regulated in much the same way as other members of the IL-1 family. For example, treatment with various microbial components (for example, PolyIC–TLR3 and flagellin–TLR5) increased IL-33 expression in human corneal epithelial cells [121] and murine macrophages [122], and pro-inflammatory cytokines including IL-3 and IL-4, and IFN-γ and TNF-α have been shown to upregulate IL-33 in a range of murine cell types [123] and human epidermal keratinocytes [114]. However, while it is clear that IL-33 can be upregulated and serves many important functions in vivo, the mechanism by which IL-33 is actively secreted from the cells is subject to ongoing debate. What is clear is that in addition to functioning as a classical cytokine, IL-33 can also function as an alarmin or danger signal that is released into the extracellular space in response to necrotic cell death or mechanical injury. For example, full-length biologically active IL-33 can be released from mechanically injured or damaged endothelial cells [111]. Given that IL-33 is constitutively expressed intracellularly in epithelial cells and endothelial cells in uninjured tissue, it is likely to function as a very efficient alarmin in vivo.

Regardless of the mechanism leading to its release/secretion, once in the extracellular environment, IL-33 has been demonstrated to have an effect on a wide range of cell types in a range of different organs [124]. IL-33 plays a role in type-2 immunity in response to helminth infection by driving the proliferation and activation (IL-13 secretion) of innate lymphoid cells (ILC2) leading to goblet cell hyperplasia and expulsion of the parasite [125–127]. The IL-33-ST2 axis has also been shown to be important in physiological remodelling of the lung following infection with influenza virus, with blockade of IL-33-ST2 resulting in severely decreased lung function, loss of airway epithelial integrity and impaired tissue remodelling [128]. IL-33-deficient mice have impaired IL-5/IL-13 secretion from ILC2, reduced eosinophil and Th2 cell recruitment in response to intranasal challenge with papain, a protease allergen [129]. Dendritic cells exposed to IL-33 secrete high levels of IL-6, TNF and IL-1β and prime naïve lymphocytes to produce Th2 cytokines in vitro while adoptive transfer of IL-33-activated dendritic cells exacerbates lung inflammation in an allergy airway inflammation model [130]. Alveolar macrophages are polarised towards an alternatively activated phenotype in response to IL-33- and ST2-deficient mice have attenuated ovalbumin-induced airway inflammation associated with a decrease in AAM differentiation [131]. Several recent publications have thoroughly reviewed the role of IL-33 in tissue injury and inflammation [124, 132, 133].

The potential of IL-33 to have a direct effect on fibroblasts has generated conflicting data. For example, IL-33 increases expression of both IL-6 and TNF-α expression in human keratinocytes [134] and has been demonstrated to induced eotaxin/CCL11 production in murine lung fibroblasts [135] and IL-6 and MCP-1 expression in murine cardiac fibroblasts [136]. However, in another study, human lung epithelial cells and endothelial cells, but not fibroblasts, were shown to express ST2 and secrete IL-8 in response to IL-33 stimulation [137]. Further work to investigate the IL-33-ST2 axis on fibroblasts is required.

IL-33 and ST2 have been demonstrated to play a role in pulmonary fibrosis in vivo [138]. In response to bleomycin challenge, mice show a substantial accumulation of IL-33-positive cells in the lung and full-length IL-33 can potentiate bleomycin-induced inflammation and fibrosis, potentially by upregulating expression of TGF-β and other non-Th2 cytokines [139]. A subsequent study demonstrated that intratracheal instillation of lentivirus expressing soluble ST2 significantly attenuated pulmonary inflammatory cell infiltration and fibrotic changes and markedly reduced the levels of inflammatory cytokines and TGF-β1 in BAL in mice challenged with bleomycin [140]. Li et al. demonstrated that ST2-deficient mice have attenuated bleomycin-induced lung fibrosis, and this phenomenon could be recreated by IL-33 neutralising antibody treatment [141]. Mechanistically, the authors demonstrate that although IL-33 is constitutively expressed in epithelial cells, it is induced in macrophages in response to bleomycin challenge and that depletion of alveolar macrophages using clodronate liposomes abolished both bleomycin-induced lung fibrosis and IL-33-exacerbated lung fibrosis. IL-33 was shown to polarise M2 macrophages to produce IL-13 and TGF-β1 and induced the expansion of type 2 ILC2s to produce IL-13 in vitro and in vivo. Furthermore, ILC2s were almost completely absent in ST2-deficient mice, and adoptive transfer of ILC2s led to exacerbation of lung inflammation and fibrosis compared to bleomycin challenge alone [141]. Patients with idiopathic pulmonary fibrosis have also been reported to have elevated levels of IL-33 in BAL and lung tissue compared to healthy controls. Significantly, Western blotting was used to determine the relative quantities of full-length and mature IL-33, and higher levels of the mature, more biologically isoform of IL-33 was seen in IPF patients [142]. In addition, serum IL-33 levels are elevated in patients with systemic sclerosis and correlated with the extent of skin sclerosis and the severity of pulmonary fibrosis [143].

Expression of IL-33 and ST2 were markedly enhanced in the lung tissue of mice challenged with cigarette smoke and were accompanied by increased neutrophil and macrophage infiltration and expression of inflammatory cytokines and chemokines. Importantly, all pathological changes were attenuated by a neutralising anti-IL-33 antibody suggesting that IL-33 plays a critical role in cigarette-smoke-mediated airway inflammation [144]. A subsequent study identified that IL-33 is expressed in bronchial endothelial cells and peripheral blood mononuclear cells in response to cigarette smoke challenge in mice and that challenge of peripheral blood mononuclear cells with IL-33 enhanced cigarette-smoke-induced inflammatory cytokine release [145]. Kearley et al. used a combination of cigarette smoke and infection to model exacerbations of COPD in mice and shown that on the background of smoke exposure, exaggerated inflammation to infection is completely attenuated in the absence of IL-33 signalling. Mechanistically, the authors demonstrate that cigarette smoke upregulates epithelial-derived IL-33 and decreases ST2 expression on ILC2s while elevating ST2 expression on macrophages and NK cells. Consequently, upon viral infection, IL-33 significantly amplified type I proinflammatory responses via synergistic modulation of macrophage and NK cell function. The authors conclude that in COPD, smoke alters the lung microenvironment to facilitate an alternative IL-33-dependent exaggerated proinflammatory response to infection, exacerbating disease [146].

IL-33 and soluble ST2 are higher in plasma samples from COPD patients compared to controls, and the frequency of IL-33 expressing peripheral blood lymphocytes and neutrophils as well as the expression of IL-33 in bronchial epithelial cells is increased in COPD patients [147]. In another study, IL-33 expression was demonstrated to be restricted to a subset of airway basal cells with increased capacities for pluripotency [148]. Smoking is the leading cause of COPD or COPD-related inflammation, and cigarette smoke extract has been shown to accentuate the IL-33-induced expression of IL-6 and IL-8 in human bronchial epithelial cells and PBMCs [149]. In mice, cigarette-smoke-induced airway inflammation is characterised by neutrophil and macrophage infiltration and increased expression of inflammatory cytokines and mucin in the airways. Significantly, expression of IL-33 and ST2 was also increased in response to cigarette smoke, and neutralising IL-33 inhibited all pathogenic changes [144]. The potential of targeting IL-33 as a therapeutic option in COPD has recently been discussed [150].

Exposure of mice to multi-walled carbon nanotubes increased BAL total cell counts, macrophage, and neutrophil recruitment, and increased inflammation and fibrosis located proximal to the airways. In contrast, IL-33-deficient mice were protected from all pathologies suggesting that IL-33 is critical for the pulmonary toxicity induced by multi-walled carbon nanotubes [151]. IL-33 has also recently been shown to be elevated in the BAL and lung tissue of CF patients where it has been hypothesised to be involved in neutrophil recruitment [152, 153].

### IL-18 subfamily

#### IL-18 and IL-18BP

Although originally identified in 1989 as ‘IFN-γ-inducing factor’, IL-18 was reclassified as a member of the IL-1 cytokine family in 1995 following purification and molecular cloning [154]. The gene, the only member of the IL-1 cytokine family to be located on chromosome 11 in humans (chromosome 9 in mice), encodes a full-length protein of ∼24 kDa that is biologically inactive. Within the IL-1 cytokine family, IL-18 is most closely related to IL-1β and shares many common traits including cleavage by caspase-1 to a biologically active mature protein of ∼17 kDa that is actively secreted from cells [155, 156]. Extracellular mature IL-18 binds to the alpha chain of the IL-18 receptor (IL-18Rα) with low affinity. Binding to a co-receptor, the beta chain of the IL-18 receptor (IL-18Rβ), forms a high-affinity heterodimer complex with signalling activity [157]. However, unlike IL-1α and β which are active on cells in the pg/ml range, IL-18 is required in high levels of >10 ng/ml to activate responsive cells [158–160]. In addition to the high levels of IL-18 required to elicit a response, there are other levels of regulation within the IL-18 signalling pathway. For example, while IL-18Rα is expressed on numerous cell types, the expression of the IL-18Rβ co-receptor is more limited, being expressed on T cells and dendritic cells but not commonly mesenchymal cells [157]. In addition, the IL-18 binding protein (IL-18BP) is a constitutively secreted protein with very high affinity for mature IL-18 that functions by sequestering IL-18 and therefore preventing the formation of IL-18-IL-18R interactions [161, 162]. IL-18BP levels in the serum of healthy individuals are in a 20-fold molar excess [163], likely acting as a very effective regulatory mechanism.

Despite this, a role for IL-18 in many biological processes has been reported [160, 164]. IL-18 is involved in regulation of the Th1 response by modulating production of IFN-γ. For example, in synergy with either IL-12 or IL-15, which upregulates the expression of the IL-18Rβ co-receptor, IL-18 induces the production of IFN-γ from T cells [165] and treatment of NK cells with IL-18 induced a CD83 + CCR7+ helper phenotype characterised by high ability to produce IFN-γ [166]. Reducing IL-18 activity using IL-18 vaccination, IL-18 neutralising antibodies, IL-18BP, or performing experiments in IL-18 deficient mice has been shown to reduce disease severity in a number of animal models including dextran sulphate sodium (DSS)-induced colitis [167, 168], collagen-induced arthritis [169, 170] and allergic airway hyperresponsiveness [171]. Readers are directed to the excellent review by Novick et al. that further highlights the many disease processes IL-18 and IL-18BP contribute to [160].

The role of the IL-18-IL-18R signalling pathway in fibrosis is unclear with both pro- and anti-fibrotic effects suggested in the literature. For example, IL-18 deficient mice were shown to have increased mortality, lung injury, and leukocyte infiltration in the BAL in response to bleomycin challenge compared to wild-type mice, and prophylactic treatment of wild-type mice with IL-18 prior to bleomycin challenge reduced lung injury and fibrosis suggesting that IL-18 plays a protective role against bleomycin-induced lung fibrosis [172]. However, a subsequent study by Hoshino et al. demonstrated that IL-18 and IL-18Rα are elevated in the lungs of patients with bleomycin-induced lethal lung injury and in the lungs of mice following bleomycin challenge and that IL-18- and IL-18Rα-deficient mice are protected from bleomycin-induced lung fibrosis, suggesting a pro-fibrotic role for IL-18 [173]. Furthermore, overexpression of IL-18 in mice drives inflammatory cell accumulation (primarily CD8^+^ T cells, macrophages, neutrophils and eosinophils) resulting in severe emphysema and airway fibrosis [174, 175]. In summary, IL-18 has been reported to have pro-fibrotic actions in vivo in the majority of studies. In agreement with this, several studies have demonstrated a direct effect for IL-18 on mesenchymal cells. IL-18 enhanced the production of angiogenic factors and key regulators of osteoclastogenesis in synovial tissue fibroblasts [176, 177]. Stimulation of cardiac fibroblasts with IL-18 promotes a pro-fibrotic response characterised by increased expression of collagen I/III and periostin, increased proliferation and increased migration [178]. IL-18 levels in serum and BAL of patients with IPF were higher than those in control subjects, and while IL-18Rα was expressed in bronchial epithelium and alveolar macrophages in control subjects, it was found to be strongly expressed in interstitial cells, especially the fibroblastic foci, in patients with IPF [179]. Conversely, a recent study by Lasithiotaki et al. failed to demonstrate a difference in IL-18 levels in BAL of patients with IPF compared to a control group but did show a significant increase in IL-18 levels in BAL of patients with rheumatoid arthritis–usual interstitial pneumonia [97].

Cigarette-smoke-induced inflammation and emphysema in the lung of wild-type mice were also associated with an increase in IL-18 expression. Importantly, IL-18Rα-deficient mice are protected from cigarette-smoke-induced inflammation and emphysema [180]. Clinically, IL-18 was shown to be elevated in pulmonary macrophages from patients with COPD, and levels of serum and circulating IL-18 have also been shown to be increased in patients with COPD [180–183]. Furthermore, there is a negative correlation between serum IL-18 level and the predicted forced expiratory volume in 1 s in patients with COPD suggesting that overproduction of IL-18 in the lungs may be involved in disease pathogenesis [184].

#### IL-37

In 2000, several independent groups described IL-37 following expression sequence tag (EST) database screening and sequencing of the IL-1 gene cluster on human (no mouse homologues have been described) chromosome 2 [185–188]. There are five spliced variants and isoforms of IL-37, termed IL-37a-e, although isoform IL-37b has the most complete set of exons and is the best characterised. IL-37 has been shown to bind to IL-18Rα, although the affinity for the receptor is lower than IL-18 and without any effect on IFN-γ production induced by IL-18 [189–191]. More recently, SIGIRR has been shown to be indispensable for IL-37 to carry out its anti-inflammatory effect [192]. IL-37 has also been demonstrated to bind to the natural inhibitor of IL-18 activity, the IL-18 binding protein (IL-18BP), leading to an enhancement of IL-18BPs ability to inhibit IFN-γ production in response to IL-18 stimulation [162, 191]. Intracellular modes of action for IL-37 have also been demonstrated. For example, when overexpressed in epithelial cells or macrophages, IL-37 almost completely suppressed the production of pro-inflammatory cytokines whereas the silencing of the IL-37 gene increased the abundance of these cytokines in human blood cells suggesting that IL-37 may possess anti-inflammatory properties [193]. Generation of an IL-37 overexpressing transgenic mouse has allowed the investigation of the role of IL-37 in vivo. For example, IL-37 transgenic mice have decreased levels of circulating cytokines, are protected from lipopolysaccharide-induced shock and show improved lung and kidney function as well as reduced liver damage in response to lipopolysaccharide [193].

Mechanistically, caspase-1-mediated cleavage to the mature form of IL-37 is required for translocation to the nucleus. In RAW macrophages, stable transfection with IL-37 substantially reduced (72–98 %) LPS-stimulated TNF-α, IL-1α and IL-6 [194]. This inhibitory effect of IL-37 is linked to binding to the Smad3 transcription factor as inhibition of Smad3 reversed the inhibition of IL-6 expression in RAW macrophages [195, 196]. The contribution of the IL-37-Smad3 interaction was confirmed in vivo using IL-37 transgenic mice that had been pretreated intranasally with siRNA specific to Smad3 prior to intranasal challenge with LPS. The reduction in cytokines in the lungs in IL-37 transgenic mice was reversed following Smad3 knockdown [193]. IL-37 transgenic mice also exhibited reduced clinical disease scores and histological evidence of colitis in the DSS model of induced colitis [197], suppressed contact hypersensitivity in response to hapten antigen 2,4-dinitrofluorobenzene by inducing tolerogenic dendritic cells [198] and are protected from myocardial and hepatic ischemic damage [199, 200]. Furthermore, recombinant human IL-37 injected intraperitoneally into mice markedly reduced NLRP3-dependent neutrophil recruitment and IL-1β production and mitigated lung inflammation and damage in response to intranasal infection with live *Aspergillus fumigatus* conidia suggesting that IL-37 functions as a broad spectrum inhibitor of the innate response to infection-mediated inflammation [201]. Excellent recent reviews further describe the anti-inflammatory action of IL-37 [185, 202].

The CLARA childhood asthma study revealed reduced mRNA expression of IL-37 in children with allergic asthma and, thus, pointed toward an implication of this cytokine for human asthma pathogenesis [203]. In addition, IL-37 levels in serum and induced sputum were lower in asthma patients compared to healthy controls and levels of IL-37 correlated with disease severity suggesting a potential protective effect [204]. A subsequent study demonstrated that IL-37 production from PBMCs was lower in allergic asthmatic compared to healthy children. The authors go on to demonstrate that intranasal IL-37 ablated airway inflammation, mucus hyperproduction and airway hyperresponsiveness in response to ovalbumin challenge via an IL-18Rα/GIGIRR-dependent pathway [205]. In contrast, level of plasma and serum IL-37 in patients with active pulmonary tuberculosis was significantly higher than that in healthy controls but recovered after treatment [206, 207]. Mechanistically, the authors demonstrate that IL-37 inhibited the production of pro-inflammatory cytokines and induced macrophages toward an M2-like phenotype [207]. IL-37 has been shown to be increased in the bronchial mucosa in COPD patients compared to control healthy smokers and non-smokers [208].

Although the exact role played by IL-37 in fibrosis is yet to be fully elucidated, it is clear that IL-37 functions as a broad-spectrum inhibitor of the innate response to infection-mediated inflammation and could be considered to be therapeutic in reducing the pulmonary damage due to non-resolving infection and disease.

### IL-36 subfamily

#### IL-36 and IL-36Ra

The IL-36 cytokine subfamily consists of three agonists, IL-36α, IL-36β and IL-36γ, which bind to IL-36R (IL-1Rrp2) and employ IL-1RAcP as a co-receptor, and a receptor antagonist, IL-36Ra, which binds to IL-36R to inhibit IL-1RAcP recruitment and the formation of a functional signalling complex [36, 188, 209, 210]. Although IL-1RAcP is shared with IL-1α, IL-1β and IL-33, the IL-36R is exclusively employed by IL-36 cytokines. Similar to other IL-1 cytokines, IL-36 cytokines require N-terminal cleavage to achieve full bioactivity (∼1000–10,000-fold increase compared to non-truncated form). Interestingly, although IL-36Ra shares 52 % homology with IL-1Ra, the antagonistic activity of IL-36Ra is uniquely dependent on post translational modification, specifically the removal of its N-terminal methionine [211]. The amino acid sequence at the truncation sites for IL-36 cytokines share little homology and do not resemble classical caspase-1 cleavage sites. Moreover, no cleavage of IL-36α is observed in bone-marrow-derived macrophages with confirmed LPS/ATP-induced caspase-1 activation suggesting that the protease(s) responsible for cleavage of IL-36 cytokines are still to be determined [211–213].

IL-36α, IL-36β and IL-36γ have been shown to have a restricted expression pattern with keratinocytes, bronchial epithelial cells, brain tissue and monocytes/macrophages as the primary sites of expression although T lymphocytes, peripheral blood lymphocytes and γδ T cells can also express IL-36 cytokines in response to a range of stimuli [213–217]. Studies in Jurkat cells transfected to express IL-36R or in a mammary epithelial cell line that naturally expresses IL-36R (NCI/ADR-RES) demonstrated that IL-36α, IL-36β and IL-36γ directly activate NF-kappaB [218] as well as MAPKs, JNK and ERK1/2 leading to the downstream activation of an IL-8 promoter reporter and the secretion of IL-6 [219]. For all molecules, blocking the IL-36R or the IL-1RAcP co-receptor inhibited the downstream effects confirming the importance of the IL-36R-IL-1RAcP complex [219].

IL-36 cytokines have also been shown to exert effects on immune cells, particularly dendritic cells. For example, mouse bone-marrow-derived dendritic cells upregulate key markers of activation (CD80, CD86 and MHCII) and produce IL-6, IL-12 and IL-23 upon stimulation with IL-36 agonists via an IL-36R-dependent pathway [215]. IL-36 has also been demonstrated to play a role in T cell polarisation by synergising with IL-12 to drive the in vitro differentiation of Th0 cells into IFN-γ + Th1 cells and to induce the production of IL-17 from murine CD4+ cells [215, 220]. Human bronchial epithelial cells stimulated with TNF, IL-17, IL-1β or double-stranded (ds)-RNA upregulated the intracellular expression of IL-36α and promoted the release of IL-36γ. Furthermore, lung fibroblasts were demonstrated to express IL-36R and IL-36RAcP and stimulation of lung fibroblasts with IL-36γ leads to the activation of MAPKs and NF-kappaB and the downstream expression of neutrophil chemokines (IL-8, CXCL3) and Th17 chemokines (CCL20). The data suggests that viral infection and/or selected cytokines from Th17 cells or inflammatory cells may drive neutrophil recruitment via IL-36γ-dependent activation of lung fibroblasts [221]. Furthermore, challenge of human bronchial epithelial cells with infectious agents such as *Pseudomonas aeruginosa* or rhinovirus has been shown to induce the expression of IL-36γ in vitro [222, 223], and intranasal challenge of mice with either IL-36α or IL-36γ induces a rapid neutrophilia [224, 225]. This data has led to the suggestion that IL-36γ released from epithelial cells may contribute to neutrophil recruitment during rhinovirus-induced exacerbation of asthma [226]. Similarly, challenge of human bronchial epithelial cells with cigarette smoke extract induced expression of IL-36α, IL-36β and IL-36γ [227]. Cigarette smoke is a causative agent for COPD, and the disease is characterised by neutrophilic accumulation in the airways. It is therefore possible that IL-36 released from epithelial cells in response to cigarette smoke challenge could contribute to the recruitment/accumulation of neutrophils seen in patients with COPD [226, 227].

Human synovial fibroblasts express IL-36R and produce pro-inflammatory mediators in response to stimulation with recombinant IL-36β, albeit to a lower magnitude than other IL-1 cytokines. In contrast, although IL-36β gene expression was elevated in response to challenge with TNF-α and IL-1β, the IL-36β protein was undetectable suggesting that fibroblasts are unlikely to be a source of IL-36β but may instead act as responsive cells. However, similar levels of IL-36β protein were detected in serum and synovial fluid isolated from healthy donors and patients with rheumatoid arthritis, osteoarthritis or septic shock, and levels of IL-36β failed to correlate with inflammation [228]. Additionally, blocking IL-36R or IL-36α, both found to be elevated in inflamed knee joints of TNF-induced arthritic mice compared to wild-type controls, had no effect on clinical onset, pattern of disease or histological evidence of arthritis suggesting that IL-36 cytokines do not affect the development of inflammatory arthritis in vivo [229], potentially due to redundancy with other members of the IL-1 family such as IL-1, a dominant cytokine in rodent arthritis models [226]. Excellent reviews further describing the biology of IL-36 cytokines have recently been published [213, 226].

There has been very little research looking at the expression of IL-36 cytokines in human disease. IL-36γ mRNA is increased in biopsies from patients with recurrent respiratory papillomas, and expression levels correlate with disease severity [230], and systemic IL-36α levels are decreased during acute exacerbation episodes in patients with COPD [231]. Further investigation is required to determine the disease relevance of these proteins and to identify a potential role in fibrosis.

#### IL-38

IL-38 was cloned and identified as a member of the IL-1 family in 2001 [232, 233]. The IL-38 gene is located on chromosome 2, between the genes encoding IL-1Ra and IL-36Ra [234], and shares 41 and 43 % homology with IL-1Ra and IL-36Ra respectively [233]. IL-38 is a 152-amino-acid protein without either a signal peptide or a caspase-1 cleavage site and is expressed in a wide range of tissues including foetal liver, salivary glands, spleen, thymus, tonsil and skin [48, 232, 233, 235]. An initial study reported that IL-38 was able to bind to IL-1R1, albeit with lower affinity than IL-1α, IL-1β or IL-1Ra [232]. However, a subsequent study has identified that IL-38 binds exclusively to IL-36R and has an antagonistic effect similar to IL-36Ra. Specifically, the authors demonstrated that *Candida albicans*-induced production of IL-22 and IL-17 in peripheral blood mononuclear cells could be reduced by IL-38 and that the level of reduction was similar to that achieved with IL-36Ra [236]. IL-38 has been shown to be elevated in the salivary glands of patients with primary Sjögren’s syndrome, and IL-38 polymorphisms are associated with psoriatic arthritis and ankylosing spondylitis [235, 237, 238]. However, to date, there is no literature regarding a role for IL-38 in fibrosis, and further work is required to determine its relevance in vivo. A recent review by Yuan et al. nicely summarises the known roles of IL-38 [239].

### Future perspective

The complexity of the IL-1 cytokine family and the body of literature relating to it continue to grow. In this review, I have attempted to provide a brief overview of the current understanding of the IL-1 cytokine family in inflammation and fibrosis in the lung while highlighting some recent key observations and review articles. However, there are still numerous outstanding questions that need to be answered. For example, although the role of some members of the IL-1 cytokine family in fibrosis is well established (IL-1α, IL-1β, IL-33), leading to compelling arguments for these molecules as potential therapeutic targets, the role of others (IL-36, IL-37, IL-38) remains to be elucidated. As we begin to understand more about the more recently described members of the IL-1 cytokine family, we are likely to identify potential new targets for consideration as innovative therapies. Furthermore, the same receptors can be employed by several members of the IL-1 cytokine family (for example, IL-1RAcP is shared by IL-1α, IL-1β, IL33 and IL-36), highlighting likely redundancy. Given the clear importance of IL-1α, IL-1β and IL-33 in lung fibrosis and the likely compensatory action provided by members of the IL-1 cytokine family in vivo, targeting the shared receptors (for example, IL-1RAcP) may be more effective. Further research to establish the potential benefits of targeting these shared receptors or simultaneously blocking multiple member of the IL-1 cytokine family in vivo is required.

Despite these outstanding questions, there is already a compelling body of preclinical evidence to support the potential of targeting IL-1 family cytokines in inflammatory and fibrotic diseases, and this has resulted in a number of compounds being evaluated as therapeutic options in a range of inflammatory and fibrotic diseases. Notable success has been achieved with Anakinra in the treatment of rheumatoid arthritis and other chronic inflammatory diseases such as gouty arthritis [240] and cryopyrin-associated periodic syndrome (CAPS) [241]. In addition, there are several compounds with mechanism of action against IL-1 family cytokines and the inflammasome currently under investigation in a range of diseases such as P2X_7_ receptor antagonists (AZD9056 - 2005-004110-32) and an anti-IL-1RI monoclonal antibody (MEDI8968 - NCT01448850). Furthermore, there are other IL-1α and IL-1β targeting agents such as Xilonix, canakinumab, rilonacept, gevokizumab and AMG108 currently under investigation for other inflammatory diseases that could also be worthwhile exploring for the treatment of chronic respiratory inflammatory disorders [241, 242].

However, the slow progression of many fibrotic diseases and the lack of early quantifiable clinical endpoints make clinical trials expensive and prohibitive; therefore, the vast majority of compounds described above will not be tested in patients with fibrotic lung disease. Therefore, research to identify bio-markers (sputum, serum, plasma, BAL, urine) and imaging technologies that can accurately measure the rate of disease progression are urgently needed. Furthermore, the majority of preclinical data described in this review has been generated in 2D submerged single cell culture systems or animal models. To build preclinical confidence, more advance culture systems (ALI, 3D culture models) and ex vivo tissue culture technique such as precision cut slices should be considered [243, 244]. Similarly, the use of primary human cells isolated from diseased tissue is preferential when interrogating mechanism of inflammation and fibrosis as there is a large body of evidence to suggest that fibroblasts isolated from normal and fibrotic tissue behave differently in culture [245–248].

## References

[1] Mannino DM, Kiriz VA (2006). Changing the burden of COPD mortality. Int J Chron Obstruct Pulmon Dis.

[2] Borthwick LA, Wynn TA, Fisher AJ (1832). Cytokine mediated tissue fibrosis. Biochim Biophys Acta.

[3] Hogg JC, Chu F, Utokaparch S, Woods R, Elliott WM, Buzatu L (2004). The nature of small-airway obstruction in chronic obstructive pulmonary disease. N Engl J Med.

[4] Coward WR, Saini G, Jenkins G (2010). The pathogenesis of idiopathic pulmonary fibrosis. Ther Adv Respir Dis.

[5] Garlanda C, Dinarello CA, Mantovani A (2013). The interleukin-1 family: back to the future. Immunity.

[6] Menkin V (1943). Studies on the isolation of the factor responsible for tissue injury in inflammation. Science.

[7] Dinarello CA, Renfer L, Wolff SM (1977). Human leukocytic pyrogen: purification and development of a radioimmunoassay. Proc Natl Acad Sci U S A.

[8] Rosenwasser LJ, Dinarello CA, Rosenthal AS (1979). Adherent cell function in murine T-lymphocyte antigen recognition. IV. Enhancement of murine T-cell antigen recognition by human leukocytic pyrogen. J Exp Med.

[9] Mizel SB, Oppenheim JJ, Rosentreich DL (1978). Characterization of lymphocyte-activating factor (LAF) produced by a macrophage cell line, P388D1. II. Biochemical characterization of LAF induced by activated T cells and LPS. J Immunol.

[10] (1979) Revised nomenclature for antigen-nonspecific T cell proliferation and helper factors. J Immunol 123:2928–2929 91646

[11] Lomedico PT, Gubler U, Hellmann CP, Dukovich M, Giri JG, Pan YC (1984). Cloning and expression of murine interleukin-1 cDNA in Escherichia coli. Nature.

[12] Auron PE, Webb AC, Rosenwasser LJ, Mucci SF, Rich A, Wolff SM (1984). Nucleotide sequence of human monocyte interleukin 1 precursor cDNA. Proc Natl Acad Sci U S A.

[13] Modi WS, Masuda A, Yamada M, Oppenheim JJ, Matsushima K, O’Brien SJ (1988). Chromosomal localization of the human interleukin 1 alpha (IL-1 alpha) gene. Genomics.

[14] Dower SK, Kronheim SR, Hopp TP, Cantrell M, Deeley M, Gillis S (1986). The cell surface receptors for interleukin-1 alpha and interleukin-1 beta are identical. Nature.

[15] Wessendorf JH, Garfinkel S, Zhan X, Brown S, Maciag T (1993). Identification of a nuclear localization sequence within the structure of the human interleukin-1 alpha precursor. J Biol Chem.

[16] Werman A, Werman-Venkert R, White R, Lee JK, Werman B, Krelin Y (2004). The precursor form of IL-1alpha is an intracrine proinflammatory activator of transcription. Proc Natl Acad Sci U S A.

[17] Dinarello CA (2009). Immunological and inflammatory functions of the interleukin-1 family. Annu Rev Immunol.

[18] Martin TR, Hagimoto N, Nakamura M, Matute-Bello G (2005). Apoptosis and epithelial injury in the lungs. Proc Am Thorac Soc.

[19] Vareille M, Kieninger E, Edwards MR, Regamey N (2011). The airway epithelium: soldier in the fight against respiratory viruses. Clin Microbiol Rev.

[20] Calderon-Garciduenas L, Osnaya N, Rodriguez-Alcaraz A, Villarreal-Calderon A (1997). DNA damage in nasal respiratory epithelium from children exposed to urban pollution. Environ Mol Mutagen.

[21] Rabinovici R, Neville LF, Abdullah F, Phillip DR, Vernick J, Fong KL (1995). Aspiration-induced lung injury: role of complement. Crit Care Med.

[22] Pauwels NS, Bracke KR, Dupont LL, Van Pottelberge GR, Provoost S, Vanden Berghe T (2011). Role of IL-1alpha and the Nlrp3/caspase-1/IL-1beta axis in cigarette smoke-induced pulmonary inflammation and COPD. Euro Respir J.

[23] Kosmider B, Messier EM, Chu HW, Mason RJ (2011). Human alveolar epithelial cell injury induced by cigarette smoke. PLoS One.

[24] Aoshiba K, Nagai A (2003). Oxidative stress, cell death, and other damage to alveolar epithelial cells induced by cigarette smoke. Tob Induc Dis.

[25] Cohen I, Rider P, Carmi Y, Braiman A, Dotan S, White MR (2010). Differential release of chromatin-bound IL-1alpha discriminates between necrotic and apoptotic cell death by the ability to induce sterile inflammation. Proc Natl Acad Sci U S A.

[26] Luheshi NM, McColl BW, Brough D (2009). Nuclear retention of IL-1 alpha by necrotic cells: a mechanism to dampen sterile inflammation. Eur J Immunol.

[27] Chen CJ, Kono H, Golenbock D, Reed G, Akira S, Rock KL (2007). Identification of a key pathway required for the sterile inflammatory response triggered by dying cells. Nat Med.

[28] Rider P, Carmi Y, Guttman O, Braiman A, Cohen I, Voronov E (2011). IL-1alpha and IL-1beta recruit different myeloid cells and promote different stages of sterile inflammation. J Immunol.

[29] Dagvadorj J, Shimada K, Chen S, Jones HD, Tumurkhuu G, Zhang W (2015). Lipopolysaccharide induces alveolar macrophage necrosis via CD14 and the P2X7 receptor leading to interleukin-1alpha release. Immunity.

[30] Yazdi AS, Guarda G, Riteau N, Drexler SK, Tardivel A, Couillin I (2010). Nanoparticles activate the NLR pyrin domain containing 3 (Nlrp3) inflammasome and cause pulmonary inflammation through release of IL-1alpha and IL-1beta. Proc Natl Acad Sci U S A.

[31] Sung SS, Walters JA (1991). Increased cyclic AMP levels enhance IL-1 alpha and IL-1 beta mRNA expression and protein production in human myelomonocytic cell lines and monocytes. J Clin Invest.

[32] Brody DT, Durum SK (1989). Membrane IL-1: IL-1 alpha precursor binds to the plasma membrane via a lectin-like interaction. J Immunol.

[33] Shreeniwas R, Koga S, Karakurum M, Pinsky D, Kaiser E, Brett J (1992). Hypoxia-mediated induction of endothelial cell interleukin-1 alpha. An autocrine mechanism promoting expression of leukocyte adhesion molecules on the vessel surface. J Clin Invest.

[34] Rider P, Carmi Y, Voronov E, Apte RN (2013). Interleukin-1alpha. Semin Immunol.

[35] Piccioli P, Rubartelli A (2013). The secretion of IL-1beta and options for release. Semin Immunol.

[36] Dinarello CA (2011). Interleukin-1 in the pathogenesis and treatment of inflammatory diseases. Blood.

[37] Martinon F, Burns K, Tschopp J (2002). The inflammasome: a molecular platform triggering activation of inflammatory caspases and processing of proIL-beta. Mol Cell.

[38] Fantuzzi G, Ku G, Harding MW, Livingston DJ, Sipe JD, Kuida K (1997). Response to local inflammation of IL-1 beta-converting enzyme- deficient mice. J Immunol.

[39] Joosten LA, Netea MG, Fantuzzi G, Koenders MI, Helsen MM, Sparrer H (2009). Inflammatory arthritis in caspase 1 gene-deficient mice: contribution of proteinase 3 to caspase 1-independent production of bioactive interleukin-1beta. Arthritis Rheum.

[40] Rubartelli A, Cozzolino F, Talio M, Sitia R (1990). A novel secretory pathway for interleukin-1 beta, a protein lacking a signal sequence. EMBO J.

[41] Andrei C, Dazzi C, Lotti L, Torrisi MR, Chimini G, Rubartelli A (1999). The secretory route of the leaderless protein interleukin 1beta involves exocytosis of endolysosome-related vesicles. Mol Biol Cell.

[42] Andrei C, Margiocco P, Poggi A, Lotti LV, Torrisi MR, Rubartelli A (2004). Phospholipases C and A2 control lysosome-mediated IL-1 beta secretion: implications for inflammatory processes. Proc Natl Acad Sci U S A.

[43] MacKenzie A, Wilson HL, Kiss-Toth E, Dower SK, North RA, Surprenant A (2001). Rapid secretion of interleukin-1beta by microvesicle shedding. Immunity.

[44] Bianco F, Pravettoni E, Colombo A, Schenk U, Moller T, Matteoli M (2005). Astrocyte-derived ATP induces vesicle shedding and IL-1 beta release from microglia. J Immunol.

[45] Qu Y, Franchi L, Nunez G, Dubyak GR (2007). Nonclassical IL-1 beta secretion stimulated by P2X7 receptors is dependent on inflammasome activation and correlated with exosome release in murine macrophages. J Immunol.

[46] Bergsbaken T, Fink SL, Cookson BT (2009). Pyroptosis: host cell death and inflammation. Nat Rev Microbiol.

[47] Eisenberg SP, Brewer MT, Verderber E, Heimdal P, Brandhuber BJ, Thompson RC (1991). Interleukin 1 receptor antagonist is a member of the interleukin 1 gene family: evolution of a cytokine control mechanism. Proc Natl Acad Sci U S A.

[48] Palomo J, Dietrich D, Martin P, Palmer G, Gabay C (2015). The interleukin (IL)-1 cytokine family - Balance between agonists and antagonists in inflammatory diseases. Cytokine.

[49] Dripps DJ, Brandhuber BJ, Thompson RC, Eisenberg SP (1991). Interleukin-1 (IL-1) receptor antagonist binds to the 80-kDa IL-1 receptor but does not initiate IL-1 signal transduction. J Biol Chem.

[50] Schreuder H, Tardif C, Trump-Kallmeyer S, Soffientini A, Sarubbi E, Akeson A (1997). A new cytokine-receptor binding mode revealed by the crystal structure of the IL-1 receptor with an antagonist. Nature.

[51] McMahan CJ, Slack JL, Mosley B, Cosman D, Lupton SD, Brunton LL (1991). A novel IL-1 receptor, cloned from B cells by mammalian expression, is expressed in many cell types. EMBO J.

[52] Kuhn PH, Marjaux E, Imhof A, De Strooper B, Haass C, Lichtenthaler SF (2007). Regulated intramembrane proteolysis of the interleukin-1 receptor II by alpha-, beta-, and gamma-secretase. J Biol Chem.

[53] Symons JA, Young PR, Duff GW (1995). Soluble type II interleukin 1 (IL-1) receptor binds and blocks processing of IL-1 beta precursor and loses affinity for IL-1 receptor antagonist. Proc Natl Acad Sci U S A.

[54] Yamaki M, Sugiura K, Muro Y, Shimoyama Y, Tomita Y (2010). Epidermal growth factor receptor tyrosine kinase inhibitors induce CCL2 and CCL5 via reduction in IL-1R2 in keratinocytes. Exp Dermatol.

[55] Suwara MI, Green NJ, Borthwick LA, Mann J, Mayer-Barber KD, Barron L (2014). IL-1alpha released from damaged epithelial cells is sufficient and essential to trigger inflammatory responses in human lung fibroblasts. Mucosal Immunol.

[56] Tracy EC, Bowman MJ, Henderson BW, Baumann H (2012). Interleukin-1alpha is the major alarmin of lung epithelial cells released during photodynamic therapy to induce inflammatory mediators in fibroblasts. Br J Cancer.

[57] Lindroos PM, Coin PG, Badgett A, Morgan DL, Bonner JC (1997). Alveolar macrophages stimulated with titanium dioxide, chrysotile asbestos, and residual oil fly ash upregulate the PDGF receptor-alpha on lung fibroblasts through an IL-1beta-dependent mechanism. Am J Respir Cell Mol Biol.

[58] Witowski J, Thiel A, Dechend R, Dunkel K, Fouquet N, Bender TO (2001). Synthesis of C-X-C and C-C chemokines by human peritoneal fibroblasts: induction by macrophage-derived cytokines. Am J Pathol.

[59] Postlethwaite AE, Raghow R, Stricklin GP, Poppleton H, Seyer JM, Kang AH (1988). Modulation of fibroblast functions by interleukin 1: increased steady-state accumulation of type I procollagen messenger RNAs and stimulation of other functions but not chemotaxis by human recombinant interleukin 1 alpha and beta. J Cell Biol.

[60] Kahari VM, Heino J, Vuorio E (1987). Interleukin-1 increases collagen production and mRNA levels in cultured skin fibroblasts. Biochim Biophys Acta.

[61] Xiao H, Ji AM, Li ZL, Song XD, Su D, Chen AH (2008). Interleukin-1beta inhibits collagen synthesis and promotes its decomposition in cultured cardiac fibroblasts. Sheng Li Xue Bao.

[62] Bhatnagar R, Penfornis H, Mauviel A, Loyau G, Saklatvala J, Pujol JP (1986). Interleukin-1 inhibits the synthesis of collagen by fibroblasts. Biochem Int.

[63] Doerner AM, Zuraw BL (2009). TGF-beta1 induced epithelial to mesenchymal transition (EMT) in human bronchial epithelial cells is enhanced by IL-1beta but not abrogated by corticosteroids. Respir Res.

[64] Borthwick LA, McIlroy EI, Gorowiec MR, Brodlie M, Johnson GE, Ward C (2010). Inflammation and epithelial to mesenchymal transition in lung transplant recipients: role in dysregulated epithelial wound repair. Am J Transplant.

[65] Rock JR, Barkauskas CE, Cronce MJ, Xue Y, Harris JR, Liang J (2011). Multiple stromal populations contribute to pulmonary fibrosis without evidence for epithelial to mesenchymal transition. Proc Natl Acad Sci U S A.

[66] Taura K, Miura K, Iwaisako K, Osterreicher CH, Kodama Y, Penz-Osterreicher M (2010). Hepatocytes do not undergo epithelial-mesenchymal transition in liver fibrosis in mice. Hepatology.

[67] Kolb M, Margetts PJ, Anthony DC, Pitossi F, Gauldie J (2001). Transient expression of IL-1beta induces acute lung injury and chronic repair leading to pulmonary fibrosis. J Clin Invest.

[68] Gasse P, Mary C, Guenon I, Noulin N, Charron S, Schnyder-Candrian S (2007). IL-1R1/MyD88 signaling and the inflammasome are essential in pulmonary inflammation and fibrosis in mice. J Clin Invest.

[69] Wilson MS, Madala SK, Ramalingam TR, Gochuico BR, Rosas IO, Cheever AW (2010). Bleomycin and IL-1beta-mediated pulmonary fibrosis is IL-17A dependent. J Exp Med.

[70] Wilmott RW, Kitzmiller JA, Fiedler MA, Stark JM (1998). Generation of a transgenic mouse with lung-specific overexpression of the human interleukin-1 receptor antagonist protein. Am J Respir Cell Mol Biol.

[71] Mulrooney N, Jobe AH, Ikegami M (2004). Lung inflammatory responses to intratracheal interleukin-1alpha in ventilated preterm lambs. Pediatr Res.

[72] Hybertson BM, Jepson EK, Allard JD, Cho OJ, Lee YM, Huddleston JR (2003). Transforming growth factor beta contributes to lung leak in rats given interleukin-1 intratracheally. Exp Lung Res.

[73] Guo JL, Gu NL, Chen J, Shi TM, Zhou Y, Rong Y (2013). Neutralization of interleukin-1 beta attenuates silica-induced lung inflammation and fibrosis in C57BL/6 mice. Arch Toxicol.

[74] Ma Y, Thornton S, Boivin GP, Hirsh D, Hirsch R, Hirsch E (1998). Altered susceptibility to collagen-induced arthritis in transgenic mice with aberrant expression of interleukin-1 receptor antagonist. Arthritis Rheum.

[75] Piguet PF, Vesin C, Grau GE, Thompson RC (1993). Interleukin 1 receptor antagonist (IL-1ra) prevents or cures pulmonary fibrosis elicited in mice by bleomycin or silica. Cytokine.

[76] Fleischmann RM, Tesser J, Schiff MH, Schechtman J, Burmester GR, Bennett R (2006). Safety of extended treatment with anakinra in patients with rheumatoid arthritis. Ann Rheum Dis.

[77] Botelho FM, Bauer CM, Finch D, Nikota JK, Zavitz CC, Kelly A (2011). IL-1alpha/IL-1R1 expression in chronic obstructive pulmonary disease and mechanistic relevance to smoke-induced neutrophilia in mice. PLoS One.

[78] Gasse P, Riteau N, Charron S, Girre S, Fick L, Petrilli V (2009). Uric acid is a danger signal activating NALP3 inflammasome in lung injury inflammation and fibrosis. Am J Respir Crit Care.

[79] Hornung V, Bauernfeind F, Halle A, Samstad EO, Kono H, Rock KL (2008). Silica crystals and aluminum salts activate the NALP3 inflammasome through phagosomal destabilization. Nat Immunol.

[80] Dostert C, Petrilli V, Van Bruggen R, Steele C, Mossman BT, Tschopp J (2008). Innate immune activation through Nalp3 inflammasome sensing of asbestos and silica. Science.

[81] dos Santos G, Rogel MR, Baker MA, Troken JR, Urich D, Morales-Nebreda L (2015). Vimentin regulates activation of the NLRP3 inflammasome. Nat Commun.

[82] Sethi JM, Rochester CL (2000). Smoking and chronic obstructive pulmonary disease. Clin Chest Med.

[83] Kuschner WG, D’Alessandro A, Wong H, Blanc PD (1996). Dose-dependent cigarette smoking-related inflammatory responses in healthy adults. Eur Respir J.

[84] Keatings VM, Collins PD, Scott DM, Barnes PJ (1996). Differences in interleukin-8 and tumor necrosis factor-alpha in induced sputum from patients with chronic obstructive pulmonary disease or asthma. Am J Respir Crit Care Med.

[85] Wanderer AA (2008). Interleukin-1beta targeted therapy in severe persistent asthma (SPA) and chronic obstructive pulmonary disease (COPD): proposed similarities between biphasic pathobiology of SPA/COPD and ischemia-reperfusion injury. Isr Med Assoc J.

[86] Yigla M, Berkovich Y, Nagler RM (2007). Oxidative stress indices in COPD--Broncho-alveolar lavage and salivary analysis. Arch Oral Biol.

[87] Bartziokas K, Papaioannou AI, Loukides S, Papadopoulos A, Haniotou A, Papiris S (2014). Serum uric acid as a predictor of mortality and future exacerbations of COPD. Eur Respir J.

[88] Eltom S, Stevenson CS, Rastrick J, Dale N, Raemdonck K, Wong S (2011). P2X7 receptor and caspase 1 activation are central to airway inflammation observed after exposure to tobacco smoke. PLoS One.

[89] Churg A, Zhou S, Wang X, Wang R, Wright JL (2009). The role of interleukin-1beta in murine cigarette smoke-induced emphysema and small airway remodeling. Am J Respir Cell Mol Biol.

[90] dos Santos G, Kutuzov MA, Ridge KM (2012). The inflammasome in lung diseases. Am J Physiol Lung Cell Mol Physiol.

[91] Lommatzsch M, Cicko S, Muller T, Lucattelli M, Bratke K, Stoll P (2010). Extracellular adenosine triphosphate and chronic obstructive pulmonary disease. Am J Respir Crit Care Med.

[92] Cicko S, Lucattelli M, Muller T, Lommatzsch M, De Cunto G, Cardini S (2010). Purinergic receptor inhibition prevents the development of smoke-induced lung injury and emphysema. J Immunol.

[93] Barlo NP, van Moorsel CH, Korthagen NM, Heron M, Rijkers GT, Ruven HJ (2011). Genetic variability in the IL1RN gene and the balance between interleukin (IL)-1 receptor agonist and IL-1beta in idiopathic pulmonary fibrosis. Clin Exp Immunol.

[94] Ogushi F, Tani K, Endo T, Tada H, Kawano T, Asano T (2001). Autoantibodies to IL-1 alpha in sera from rapidly progressive idiopathic pulmonary fibrosis. J Med Invest.

[95] Zhang Y, Lee TC, Guillemin B, Yu MC, Rom WN (1993). Enhanced IL-1 beta and tumor necrosis factor-alpha release and messenger RNA expression in macrophages from idiopathic pulmonary fibrosis or after asbestos exposure. J Immunol.

[96] Kline JN, Schwartz DA, Monick MM, Floerchinger CS, Hunninghake GW (1993). Relative release of interleukin-1 beta and interleukin-1 receptor antagonist by alveolar macrophages. A study in asbestos-induced lung disease, sarcoidosis, and idiopathic pulmonary fibrosis. Chest.

[97] Lasithiotaki I, Giannarakis I, Tsitoura E, Samara KD, Margaritopoulos GA, Choulaki C (2016). NLRP3 inflammasome expression in idiopathic pulmonary fibrosis and rheumatoid lung. Eur Respir J.

[98] Riteau N, Gasse P, Fauconnier L, Gombault A, Couegnat M, Fick L (2010). Extracellular ATP is a danger signal activating P2X7 receptor in lung inflammation and fibrosis. Am J Respir Crit Care Med.

[99] Wollin L, Maillet I, Quesniaux V, Holweg A, Ryffel B (2014). Antifibrotic and anti-inflammatory activity of the tyrosine kinase inhibitor nintedanib in experimental models of lung fibrosis. J Pharmacol Exp Ther.

[100] Oku H, Shimizu T, Kawabata T, Nagira M, Hikita I, Ueyama A (2008). Antifibrotic action of pirfenidone and prednisolone: different effects on pulmonary cytokines and growth factors in bleomycin-induced murine pulmonary fibrosis. Eur J Pharmacol.

[101] Belperio JA, DiGiovine B, Keane MP, Burdick MD, Ying Xue Y, Ross DJ (2002). Interleukin-1 receptor antagonist as a biomarker for bronchiolitis obliterans syndrome in lung transplant recipients. Transplantation.

[102] Borthwick LA, Corris PA, Mahida R, Walker A, Gardner A, Suwara M (2013). TNFalpha from classically activated macrophages accentuates epithelial to mesenchymal transition in obliterative bronchiolitis. Am J Transplant.

[103] Borthwick LA, Suwara MI, Carnell SC, Green NJ, Mahida R, Dixon D (2015). Pseudomonas aeruginosa induced airway epithelial injury drives fibroblast activation: a mechanism in chronic lung allograft dysfunction. Am J Transplant.

[104] Schmitz J, Owyang A, Oldham E, Song Y, Murphy E, McClanahan TK (2005). IL-33, an interleukin-1-like cytokine that signals via the IL-1 receptor-related protein ST2 and induces T helper type 2-associated cytokines. Immunity.

[105] Baekkevold ES, Roussigne M, Yamanaka T, Johansen FE, Jahnsen FL, Amalric F (2003). Molecular characterization of NF-HEV, a nuclear factor preferentially expressed in human high endothelial venules. Am J Pathol.

[106] Carriere V, Roussel L, Ortega N, Lacorre DA, Americh L, Aguilar L (2007). IL-33, the IL-1-like cytokine ligand for ST2 receptor, is a chromatin-associated nuclear factor in vivo. Proc Natl Acad Sci U S A.

[107] Roussel L, Erard M, Cayrol C, Girard JP (2008). Molecular mimicry between IL-33 and KSHV for attachment to chromatin through the H2A-H2B acidic pocket. EMBO Rep.

[108] Martin MU (2013). Special aspects of interleukin-33 and the IL-33 receptor complex. Semin Immunol.

[109] Ali S, Mohs A, Thomas M, Klare J, Ross R, Schmitz ML (2011). The dual function cytokine IL-33 interacts with the transcription factor NF-kappaB to dampen NF-kappaB-stimulated gene transcription. J Immunol.

[110] Lefrancais E, Roga S, Gautier V, Gonzalez-de-Peredo A, Monsarrat B, Girard JP (2012). IL-33 is processed into mature bioactive forms by neutrophil elastase and cathepsin G. Proc Natl Acad Sci U S A.

[111] Cayrol C, Girard JP (2009). The IL-1-like cytokine IL-33 is inactivated after maturation by caspase-1. Proc Natl Acad Sci U S A.

[112] Ali S, Nguyen DQ, Falk W, Martin MU (2010). Caspase 3 inactivates biologically active full length interleukin-33 as a classical cytokine but does not prohibit nuclear translocation. Biochem Biophys Res Commun.

[113] Luthi AU, Cullen SP, McNeela EA, Duriez PJ, Afonina IS, Sheridan C (2009). Suppression of interleukin-33 bioactivity through proteolysis by apoptotic caspases. Immunity.

[114] Meephansan J, Tsuda H, Komine M, Tominaga S, Ohtsuki M (2012). Regulation of IL-33 expression by IFN-gamma and tumor necrosis factor-alpha in normal human epidermal keratinocytes. J Invest Dermatol.

[115] Hayakawa M, Hayakawa H, Matsuyama Y, Tamemoto H, Okazaki H, Tominaga S (2009). Mature interleukin-33 is produced by calpain-mediated cleavage in vivo. Biochem Biophys Res Commun.

[116] Ali S, Hubert M, Kollewe C, Bischoff SC, Falk W, Martin MU (2007). IL-1 receptor accessory protein is essential for IL-33-induced activation of T lymphocytes and mast cells. Proc Natl Acad Sci U S A.

[117] Chackerian AA, Oldham ER, Murphy EE, Schmitz J, Pflanz S, Kastelein RA (2007). IL-1 receptor accessory protein and ST2 comprise the IL-33 receptor complex. J Immunol.

[118] Hayakawa H, Hayakawa M, Kume A, Tominaga S (2007). Soluble ST2 blocks interleukin-33 signaling in allergic airway inflammation. J Biol Chem.

[119] Tago K, Noda T, Hayakawa M, Iwahana H, Yanagisawa K, Yashiro T (2001). Tissue distribution and subcellular localization of a variant form of the human ST2 gene product, ST2V. Biochem Biophys Res Commun.

[120] Smith DE (2010). IL-33: a tissue derived cytokine pathway involved in allergic inflammation and asthma. Clin Exp Allergy.

[121] Zhang L, Lu R, Zhao G, Pflugfelder SC, Li DQ (2011). TLR-mediated induction of pro-allergic cytokine IL-33 in ocular mucosal epithelium. Int J Biochem Cell Biol.

[122] Polumuri SK, Jayakar GG, Shirey KA, Roberts ZJ, Perkins DJ, Pitha PM (2012). Transcriptional regulation of murine IL-33 by TLR and non-TLR agonists. J Immunol.

[123] Zhao WH, Hu ZQ (2012). Up-regulation of IL-33 expression in various types of murine cells by IL-3 and IL-4. Cytokine.

[124] Cayrol C, Girard JP (2014). IL-33: an alarmin cytokine with crucial roles in innate immunity, inflammation and allergy. Curr Opin Immunol.

[125] Moro K, Yamada T, Tanabe M, Takeuchi T, Ikawa T, Kawamoto H (2010). Innate production of T(H)2 cytokines by adipose tissue-associated c-Kit(+)Sca-1(+) lymphoid cells. Nature.

[126] Neill DR, Wong SH, Bellosi A, Flynn RJ, Daly M, Langford TK (2010). Nuocytes represent a new innate effector leukocyte that mediates type-2 immunity. Nature.

[127] Price AE, Liang HE, Sullivan BM, Reinhardt RL, Eisley CJ, Erle DJ (2010). Systemically dispersed innate IL-13-expressing cells in type 2 immunity. Proc Natl Acad Sci U S A.

[128] Monticelli LA, Sonnenberg GF, Abt MC, Alenghat T, Ziegler CG, Doering TA (2011). Innate lymphoid cells promote lung-tissue homeostasis after infection with influenza virus. Nat Immunol.

[129] Kamijo S, Takeda H, Tokura T, Suzuki M, Inui K, Hara M (2013). IL-33-mediated innate response and adaptive immune cells contribute to maximum responses of protease allergen-induced allergic airway inflammation. J Immunol.

[130] Besnard AG, Togbe D, Guillou N, Erard F, Quesniaux V, Ryffel B (2011). IL-33-activated dendritic cells are critical for allergic airway inflammation. Eur J Immunol.

[131] Kurowska-Stolarska M, Stolarski B, Kewin P, Murphy G, Corrigan CJ, Ying S (2009). IL-33 amplifies the polarization of alternatively activated macrophages that contribute to airway inflammation. J Immunol.

[132] Molofsky AB, Savage AK, Locksley RM (2015). Interleukin-33 in tissue homeostasis, injury, and inflammation. Immunity.

[133] Theoharides TC, Petra AI, Taracanova A, Panagiotidou S, Conti P (2015). Targeting IL-33 in autoimmunity and inflammation. J Pharmacol Exp Ther.

[134] Li P, Ma H, Han D, Mou K (2015). Interleukin-33 affects cytokine production by keratinocytes in vitiligo. Clin Exp Dermatol.

[135] Kurokawa M, Matsukura S, Kawaguchi M, Ieki K, Suzuki S, Odaka M (2011). Expression and effects of IL-33 and ST2 in allergic bronchial asthma: IL-33 induces eotaxin production in lung fibroblasts. Int Arch Allergy Immunol.

[136] Zhu J, Carver W (2012). Effects of interleukin-33 on cardiac fibroblast gene expression and activity. Cytokine.

[137] Yagami A, Orihara K, Morita H, Futamura K, Hashimoto N, Matsumoto K (2010). IL-33 mediates inflammatory responses in human lung tissue cells. J Immunol.

[138] Gao Q, Li Y, Li M (2015). The potential role of IL-33/ST2 signaling in fibrotic diseases. J Leukoc Biol.

[139] Luzina IG, Kopach P, Lockatell V, Kang PH, Nagarsekar A, Burke AP (2013). Interleukin-33 potentiates bleomycin-induced lung injury. Am J Respir Cell Mol Biol.

[140] Gao Q, Li Y, Pan X, Yuan X, Peng X, Li M (2016). Lentivirus expressing soluble ST2 alleviates bleomycin-induced pulmonary fibrosis in mice. Int Immunopharmacol.

[141] Li D, Guabiraba R, Besnard AG, Komai-Koma M, Jabir MS, Zhang L (2014). IL-33 promotes ST2-dependent lung fibrosis by the induction of alternatively activated macrophages and innate lymphoid cells in mice. J Allergy Clin Immunol.

[142] Luzina IG, Pickering EM, Kopach P, Kang PH, Lockatell V, Todd NW (2012). Full-length IL-33 promotes inflammation but not Th2 response in vivo in an ST2-independent fashion. J Immunol.

[143] Yanaba K, Yoshizaki A, Asano Y, Kadono T, Sato S (2011). Serum IL-33 levels are raised in patients with systemic sclerosis: association with extent of skin sclerosis and severity of pulmonary fibrosis. Clin Rheumatol.

[144] Qiu C, Li Y, Li M, Li M, Liu X, McSharry C (2013). Anti-interleukin-33 inhibits cigarette smoke-induced lung inflammation in mice. Immunology.

[145] Wu H, Yang S, Wu X, Zhao J, Zhao J, Ning Q (2014). Interleukin-33/ST2 signaling promotes production of interleukin-6 and interleukin-8 in systemic inflammation in cigarette smoke-induced chronic obstructive pulmonary disease mice. Biochem Biophys Res Commun.

[146] Kearley J, Silver JS, Sanden C, Liu Z, Berlin AA, White N (2015). Cigarette smoke silences innate lymphoid cell function and facilitates an exacerbated type I interleukin-33-dependent response to infection. Immunity.

[147] Xia J, Zhao J, Shang J, Li M, Zeng Z, Zhao J (2015). Increased IL-33 expression in chronic obstructive pulmonary disease. Am J Physiol Lung Cell Mol Physiol.

[148] Byers DE, Alexander-Brett J, Patel AC, Agapov E, Dang-Vu G, Jin X (2013). Long-term IL-33-producing epithelial progenitor cells in chronic obstructive lung disease. J Clin Invest.

[149] Shang J, Zhao J, Wu X, Xu Y, Xie J, Zhao J (2015). Interleukin-33 promotes inflammatory cytokine production in chronic airway inflammation. Biochem Cell Biol.

[150] Barnes PJ (2015). Therapeutic approaches to asthma-chronic obstructive pulmonary disease overlap syndromes. J Allergy Clin Immunol.

[151] Wang X, Shannahan JH, Brown JM (2014). IL-33 modulates chronic airway resistance changes induced by multi-walled carbon nanotubes. Inhal Toxicol.

[152] Tiringer K, Treis A, Kanolzer S, Witt C, Ghanim B, Gruber S (2014). Differential expression of IL-33 and HMGB1 in the lungs of stable cystic fibrosis patients. Eur Respir J.

[153] Roussel L, Farias R, Rousseau S (2013). IL-33 is expressed in epithelia from patients with cystic fibrosis and potentiates neutrophil recruitment. J Allergy Clin Immunol.

[154] Okamura H, Nagata K, Komatsu T, Tanimoto T, Nukata Y, Tanabe F (1995). A novel costimulatory factor for gamma interferon induction found in the livers of mice causes endotoxic shock. Infect Immun.

[155] Gu Y, Kuida K, Tsutsui H, Ku G, Hsiao K, Fleming MA (1997). Activation of interferon-gamma inducing factor mediated by interleukin-1beta converting enzyme. Science.

[156] Ghayur T, Banerjee S, Hugunin M, Butler D, Herzog L, Carter A (1997). Caspase-1 processes IFN-gamma-inducing factor and regulates LPS-induced IFN-gamma production. Nature.

[157] Dinarello CA, Novick D, Kim S, Kaplanski G (2013). Interleukin-18 and IL-18 binding protein. Front Immunol.

[158] Morel JC, Park CC, Woods JM, Koch AE (2001). A novel role for interleukin-18 in adhesion molecule induction through NF kappa B and phosphatidylinositol (PI) 3-kinase-dependent signal transduction pathways. J Biol Chem.

[159] Lee JK, Kim SH, Lewis EC, Azam T, Reznikov LL, Dinarello CA (2004). Differences in signaling pathways by IL-1beta and IL-18. Proc Natl Acad Sci U S A.

[160] Novick D, Kim S, Kaplanski G, Dinarello CA (2013). Interleukin-18, more than a Th1 cytokine. Semin Immunol.

[161] Kim SH, Eisenstein M, Reznikov L, Fantuzzi G, Novick D, Rubinstein M (2000). Structural requirements of six naturally occurring isoforms of the IL-18 binding protein to inhibit IL-18. Proc Natl Acad Sci U S A.

[162] Novick D, Kim SH, Fantuzzi G, Reznikov LL, Dinarello CA, Rubinstein M (1999). Interleukin-18 binding protein: a novel modulator of the Th1 cytokine response. Immunity.

[163] Novick D, Schwartsburd B, Pinkus R, Suissa D, Belzer I, Sthoeger Z (2001). A novel IL-18BP ELISA shows elevated serum IL-18BP in sepsis and extensive decrease of free IL-18. Cytokine.

[164] Nakanishi K, Yoshimoto T, Tsutsui H, Okamura H (2001). Interleukin-18 is a unique cytokine that stimulates both Th1 and Th2 responses depending on its cytokine milieu. Cytokine Growth Factor Rev.

[165] Micallef MJ, Ohtsuki T, Kohno K, Tanabe F, Ushio S, Namba M (1996). Interferon-gamma-inducing factor enhances T helper 1 cytokine production by stimulated human T cells: synergism with interleukin-12 for interferon-gamma production. Eur J Immunol.

[166] Mailliard RB, Alber SM, Shen H, Watkins SC, Kirkwood JM, Herberman RB (2005). IL-18-induced CD83 + CCR7+ NK helper cells. J Exp Med.

[167] Siegmund B, Fantuzzi G, Rieder F, Gamboni-Robertson F, Lehr HA, Hartmann G (2001). Neutralization of interleukin-18 reduces severity in murine colitis and intestinal IFN-gamma and TNF-alpha production. Am J Physiol Regul Integr Comp Physiol.

[168] Sivakumar PV, Westrich GM, Kanaly S, Garka K, Born TL, Derry JM (2002). Interleukin 18 is a primary mediator of the inflammation associated with dextran sulphate sodium induced colitis: blocking interleukin 18 attenuates intestinal damage. Gut.

[169] Plater-Zyberk C, Joosten LA, Helsen MM, Sattonnet-Roche P, Siegfried C, Alouani S (2001). Therapeutic effect of neutralizing endogenous IL-18 activity in the collagen-induced model of arthritis. J Clin Invest.

[170] Wei XQ, Leung BP, Arthur HM, McInnes IB, Liew FY (2001). Reduced incidence and severity of collagen-induced arthritis in mice lacking IL-18. J Immunol.

[171] Maecker HT, Hansen G, Walter DM, DeKruyff RH, Levy S, Umetsu DT (2001). Vaccination with allergen-IL-18 fusion DNA protects against, and reverses established, airway hyperreactivity in a murine asthma model. J Immunol.

[172] Nakatani-Okuda A, Ueda H, Kashiwamura S, Sekiyama A, Kubota A, Fujita Y (2005). Protection against bleomycin-induced lung injury by IL-18 in mice. Am J Physiol Lung Cell Mol Physiol.

[173] Hoshino T, Okamoto M, Sakazaki Y, Kato S, Young HA, Aizawa H (2009). Role of proinflammatory cytokines IL-18 and IL-1beta in bleomycin-induced lung injury in humans and mice. Am J Respir Cell Mol Biol.

[174] Hoshino T, Kato S, Oka N, Imaoka H, Kinoshita T, Takei S (2007). Pulmonary inflammation and emphysema: role of the cytokines IL-18 and IL-13. Am J Respir Crit Care Med.

[175] Kang MJ, Choi JM, Kim BH, Lee CM, Cho WK, Choe G (2012). IL-18 induces emphysema and airway and vascular remodeling via IFN-gamma, IL-17A, and IL-13. Am J Respir Crit Care Med.

[176] Amin MA, Mansfield PJ, Pakozdi A, Campbell PL, Ahmed S, Martinez RJ (2007). Interleukin-18 induces angiogenic factors in rheumatoid arthritis synovial tissue fibroblasts via distinct signaling pathways. Arthritis Rheum.

[177] Zhang W, Cong XL, Qin YH, He ZW, He DY, Dai SM (2013). IL-18 upregulates the production of key regulators of osteoclastogenesis from fibroblast-like synoviocytes in rheumatoid arthritis. Inflammation.

[178] Fix C, Bingham K, Carver W (2011). Effects of interleukin-18 on cardiac fibroblast function and gene expression. Cytokine.

[179] Kitasato Y, Hoshino T, Okamoto M, Kato S, Koda Y, Nagata N (2004). Enhanced expression of interleukin-18 and its receptor in idiopathic pulmonary fibrosis. Am J Respir Cell Mol Biol.

[180] Kang MJ, Homer RJ, Gallo A, Lee CG, Crothers KA, Cho SJ (2007). IL-18 is induced and IL-18 receptor alpha plays a critical role in the pathogenesis of cigarette smoke-induced pulmonary emphysema and inflammation. J Immunol.

[181] Petersen AM, Penkowa M, Iversen M, Frydelund-Larsen L, Andersen JL, Mortensen J (2007). Elevated levels of IL-18 in plasma and skeletal muscle in chronic obstructive pulmonary disease. Lung.

[182] Wang J, Liu X, Xie M, Xie J, Xiong W, Xu Y (2012). Increased expression of interleukin-18 and its receptor in peripheral blood of patients with chronic obstructive pulmonary disease. COPD.

[183] Rovina N, Dima E, Gerassimou C, Kollintza A, Gratziou C, Roussos C (2009). Interleukin-18 in induced sputum: association with lung function in chronic obstructive pulmonary disease. Respir Med.

[184] Imaoka H, Hoshino T, Takei S, Kinoshita T, Okamoto M, Kawayama T (2008). Interleukin-18 production and pulmonary function in COPD. Eur Respir J.

[185] Boraschi D, Lucchesi D, Hainzl S, Leitner M, Maier E, Mangelberger D (2011). IL-37: a new anti-inflammatory cytokine of the IL-1 family. Eur Cytokine Netw.

[186] Smith DE, Renshaw BR, Ketchem RR, Kubin M, Garka KE, Sims JE (2000). Four new members expand the interleukin-1 superfamily. J Biol Chem.

[187] Busfield SJ, Comrack CA, Yu G, Chickering TW, Smutko JS, Zhou H (2000). Identification and gene organization of three novel members of the IL-1 family on human chromosome 2. Genomics.

[188] Kumar S, McDonnell PC, Lehr R, Tierney L, Tzimas MN, Griswold DE (2000). Identification and initial characterization of four novel members of the interleukin-1 family. J Biol Chem.

[189] Kumar S, Hanning CR, Brigham-Burke MR, Rieman DJ, Lehr R, Khandekar S (2002). Interleukin-1F7B (IL-1H4/IL-1F7) is processed by caspase-1 and mature IL-1F7B binds to the IL-18 receptor but does not induce IFN-gamma production. Cytokine.

[190] Pan G, Risser P, Mao W, Baldwin DT, Zhong AW, Filvaroff E (2001). IL-1H, an interleukin 1-related protein that binds IL-18 receptor/IL-1Rrp. Cytokine.

[191] Bufler P, Azam T, Gamboni-Robertson F, Reznikov LL, Kumar S, Dinarello CA (2002). A complex of the IL-1 homologue IL-1F7b and IL-18-binding protein reduces IL-18 activity. Proc Natl Acad Sci U S A.

[192] Nold-Petry CA, Lo CY, Rudloff I, Elgass KD, Li S, Gantier MP (2015). IL-37 requires the receptors IL-18Ralpha and IL-1R8 (SIGIRR) to carry out its multifaceted anti-inflammatory program upon innate signal transduction. Nat Immunol.

[193] Nold MF, Nold-Petry CA, Zepp JA, Palmer BE, Bufler P, Dinarello CA (2010). IL-37 is a fundamental inhibitor of innate immunity. Nat Immunol.

[194] Sharma S, Kulk N, Nold MF, Graf R, Kim SH, Reinhardt D (2008). The IL-1 family member 7b translocates to the nucleus and down-regulates proinflammatory cytokines. J Immunol.

[195] Grimsby S, Jaensson H, Dubrovska A, Lomnytska M, Hellman U, Souchelnytskyi S (2004). Proteomics-based identification of proteins interacting with Smad3: SREBP-2 forms a complex with Smad3 and inhibits its transcriptional activity. FEBS Lett.

[196] Bar D, Apte RN, Voronov E, Dinarello CA, Cohen S (2004). A continuous delivery system of IL-1 receptor antagonist reduces angiogenesis and inhibits tumor development. FASEB J.

[197] McNamee EN, Masterson JC, Jedlicka P, McManus M, Grenz A, Collins CB (2011). Interleukin 37 expression protects mice from colitis. Proc Natl Acad Sci U S A.

[198] Luo Y, Cai X, Liu S, Wang S, Nold-Petry CA, Nold MF (2014). Suppression of antigen-specific adaptive immunity by IL-37 via induction of tolerogenic dendritic cells. Proc Natl Acad Sci U S A.

[199] Dinarello CA, Bufler P (2013). Interleukin-37. Semin Immunol.

[200] Sakai N, Van Sweringen HL, Belizaire RM, Quillin RC, Schuster R, Blanchard J (2012). Interleukin-37 reduces liver inflammatory injury via effects on hepatocytes and non-parenchymal cells. J Gastroenterol Hepatol.

[201] Moretti S, Bozza S, Oikonomou V, Renga G, Casagrande A, Iannitti RG (2014). IL-37 inhibits inflammasome activation and disease severity in murine aspergillosis. PLoS Pathog.

[202] Quirk S, Agrawal DK (2014). Immunobiology of IL-37: mechanism of action and clinical perspectives. Expert Rev Clin Immunol.

[203] Raedler D, Ballenberger N, Klucker E, Bock A, Otto R, Prazeres da Costa O (2015). Identification of novel immune phenotypes for allergic and nonallergic childhood asthma. J Allergy Clin Immunol.

[204] Charrad R, Berraies A, Hamdi B, Ammar J, Hamzaoui K, Hamzaoui A (2016) Anti-inflammatory activity of IL-37 in asthmatic children: correlation with inflammatory cytokines TNF-alpha, IL-beta, IL-6 and IL-17A. Immunobiology 221(2):182–187. doi:10.1016/j.imbio.2015.09.009. 10.1016/j.imbio.2015.09.009 26454413

[205] Lunding L, Webering S, Vock C, Schroder A, Raedler D, Schaub B (2015). IL-37 requires IL-18Ralpha and SIGIRR/IL-1R8 to diminish allergic airway inflammation in mice. Allergy.

[206] Zhang J, Liu G, Zeng J, Wang W, Xiang W, Kong B (2015). Clinical detection and significance of plasma IL-37 in patients with active pulmonary tuberculosis. Xi Bao Yu Fen Zi Mian Yi Xue Za Zhi.

[207] Huang Z, Gao C, Chi X, Hu YW, Zheng L, Zeng T (2015). IL-37 expression is upregulated in patients with tuberculosis and induces macrophages towards an M2-like phenotype. Scand J Immunol.

[208] Di Stefano A, Caramori G, Barczyk A, Vicari C, Brun P, Zanini A (2014). Innate immunity but not NLRP3 inflammasome activation correlates with severity of stable COPD. Thorax.

[209] Sims JE, Smith DE (2010). The IL-1 family: regulators of immunity. Nat Rev Immunol.

[210] Abbatt JD, Lea AJ (1956). The incidence of leukaemia in ankylosing spondylitis treated with x-rays. Lancet.

[211] Towne JE, Renshaw BR, Douangpanya J, Lipsky BP, Shen M, Gabel CA (2011). Interleukin-36 (IL-36) ligands require processing for full agonist (IL-36alpha, IL-36beta, and IL-36gamma) or antagonist (IL-36Ra) activity. J Biol Chem.

[212] Martin U, Scholler J, Gurgel J, Renshaw B, Sims JE, Gabel CA (2009). Externalization of the leaderless cytokine IL-1F6 occurs in response to lipopolysaccharide/ATP activation of transduced bone marrow macrophages. J Immunol.

[213] Gresnigt MS, van de Veerdonk FL (2013). Biology of IL-36 cytokines and their role in disease. Semin Immunol.

[214] Li Y, Messina C, Bendaoud M, Fine DH, Schreiner H, Tsiagbe VK (2010). Adaptive immune response in osteoclastic bone resorption induced by orally administered Aggregatibacter actinomycetemcomitans in a rat model of periodontal disease. Mol Oral Microbiol.

[215] Vigne S, Palmer G, Lamacchia C, Martin P, Talabot-Ayer D, Rodriguez E (2011). IL-36R ligands are potent regulators of dendritic and T cells. Blood.

[216] Turtoi A, Brown I, Schlager M, Schneeweiss FH (2010). Gene expression profile of human lymphocytes exposed to (211)At alpha particles. Radiat Res.

[217] Yang J, Meyer M, Muller AK, Bohm F, Grose R, Dauwalder T (2010). Fibroblast growth factor receptors 1 and 2 in keratinocytes control the epidermal barrier and cutaneous homeostasis. J Cell Biol.

[218] Debets R, Timans JC, Homey B, Zurawski S, Sana TR, Lo S (2001). Two novel IL-1 family members, IL-1 delta and IL-1 epsilon, function as an antagonist and agonist of NF-kappa B activation through the orphan IL-1 receptor-related protein 2. J Immunol.

[219] Towne JE, Garka KE, Renshaw BR, Virca GD, Sims JE (2004). Interleukin (IL)-1F6, IL-1F8, and IL-1F9 signal through IL-1Rrp2 and IL-1RAcP to activate the pathway leading to NF-kappaB and MAPKs. J Biol Chem.

[220] Vigne S, Palmer G, Martin P, Lamacchia C, Strebel D, Rodriguez E (2012). IL-36 signaling amplifies Th1 responses by enhancing proliferation and Th1 polarization of naive CD4+ T cells. Blood.

[221] Chustz RT, Nagarkar DR, Poposki JA, Favoreto S, Avila PC, Schleimer RP (2011). Regulation and function of the IL-1 family cytokine IL-1F9 in human bronchial epithelial cells. Am J Respir Cell Mol Biol.

[222] Bochkov YA, Hanson KM, Keles S, Brockman-Schneider RA, Jarjour NN, Gern JE (2010). Rhinovirus-induced modulation of gene expression in bronchial epithelial cells from subjects with asthma. Mucosal Immunol.

[223] Vos JB, van Sterkenburg MA, Rabe KF, Schalkwijk J, Hiemstra PS, Datson NA (2005). Transcriptional response of bronchial epithelial cells to Pseudomonas aeruginosa: identification of early mediators of host defense. Physiol Genomics.

[224] Ramadas RA, Ewart SL, Medoff BD, LeVine AM (2011). Interleukin-1 family member 9 stimulates chemokine production and neutrophil influx in mouse lungs. Am J Respir Cell Mol Biol.

[225] Ramadas RA, Ewart SL, Iwakura Y, Medoff BD, LeVine AM (2012). IL-36alpha exerts pro-inflammatory effects in the lungs of mice. PLoS One.

[226] Gabay C, Towne JE (2015). Regulation and function of interleukin-36 cytokines in homeostasis and pathological conditions. J Leukoc Biol.

[227] Parsanejad R, Fields WR, Steichen TJ, Bombick BR, Doolittle DJ (2008). Distinct regulatory profiles of interleukins and chemokines in response to cigarette smoke condensate in normal human bronchial epithelial (NHBE) cells. J Interferon Cytokine Res.

[228] Magne D, Palmer G, Barton JL, Mezin F, Talabot-Ayer D, Bas S (2006). The new IL-1 family member IL-1F8 stimulates production of inflammatory mediators by synovial fibroblasts and articular chondrocytes. Arthritis Res Ther.

[229] Derer A, Groetsch B, Harre U, Bohm C, Towne J, Schett G (2014). Blockade of IL-36 receptor signaling does not prevent from TNF-induced arthritis. PLoS One.

[230] DeVoti JA, Rosenthal DW, Wu R, Abramson AL, Steinberg BM, Bonagura VR (2008). Immune dysregulation and tumor-associated gene changes in recurrent respiratory papillomatosis: a paired microarray analysis. Mol Med.

[231] Chen H, Wang Y, Bai C, Wang X (2012). Alterations of plasma inflammatory biomarkers in the healthy and chronic obstructive pulmonary disease patients with or without acute exacerbation. J Proteomics.

[232] Lin H, Ho AS, Haley-Vicente D, Zhang J, Bernal-Fussell J, Pace AM (2001). Cloning and characterization of IL-1HY2, a novel interleukin-1 family member. J Biol Chem.

[233] Bensen JT, Dawson PA, Mychaleckyj JC, Bowden DW (2001). Identification of a novel human cytokine gene in the interleukin gene cluster on chromosome 2q12-14. J Interferon Cytokine Res.

[234] Nicklin MJ, Barton JL, Nguyen M, FitzGerald MG, Duff GW, Kornman K (2002). A sequence-based map of the nine genes of the human interleukin-1 cluster. Genomics.

[235] Ciccia F, Accardo-Palumbo A, Alessandro R, Alessandri C, Priori R, Guggino G (2015). Interleukin-36alpha axis is modulated in patients with primary Sjogren’s syndrome. Clin Exp Immunol.

[236] van de Veerdonk FL, Stoeckman AK, Wu G, Boeckermann AN, Azam T, Netea MG (2012). IL-38 binds to the IL-36 receptor and has biological effects on immune cells similar to IL-36 receptor antagonist. Proc Natl Acad Sci U S A.

[237] Monnet D, Kadi A, Izac B, Lebrun N, Letourneur F, Zinovieva E (2012). Association between the IL-1 family gene cluster and spondyloarthritis. Ann Rheum Dis.

[238] Lea WI, Lee YH (2012). The associations between interleukin-1 polymorphisms and susceptibility to ankylosing spondylitis: a meta-analysis. Jt Bone Spine.

[239] Yuan X, Peng X, Li Y, Li M (2015). Role of IL-38 and its related cytokines in inflammation. Mediat Inflamm.

[240] Ottaviani S, Molto A, Ea HK, Neveu S, Gill G, Brunier L (2013). Efficacy of anakinra in gouty arthritis: a retrospective study of 40 cases. Arthritis Res Ther.

[241] Dinarello CA, Simon A, van der Meer JW (2012). Treating inflammation by blocking interleukin-1 in a broad spectrum of diseases. Nat Rev Drug Discov.

[242] Brusselle GG, Provoost S, Bracke KR, Kuchmiy A, Lamkanfi M (2014). Inflammasomes in respiratory disease: from bench to bedside. Chest.

[243] Haycock JW (2011). 3D cell culture: a review of current approaches and techniques. Methods Mol Biol.

[244] Morin JP, Baste JM, Gay A, Crochemore C, Corbiere C, Monteil C (2013). Precision cut lung slices as an efficient tool for in vitro lung physio-pharmacotoxicology studies. Xenobiotica.

[245] Ramos C, Montano M, Garcia-Alvarez J, Ruiz V, Uhal BD, Selman M (2001). Fibroblasts from idiopathic pulmonary fibrosis and normal lungs differ in growth rate, apoptosis, and tissue inhibitor of metalloproteinases expression. Am J Respir Cell Mol Biol.

[246] Emblom-Callahan MC, Chhina MK, Shlobin OA, Ahmad S, Reese ES, Iyer EP (2010). Genomic phenotype of non-cultured pulmonary fibroblasts in idiopathic pulmonary fibrosis. Genomics.

[247] Huang SK, Scruggs AM, McEachin RC, White ES, Peters-Golden M (2014). Lung fibroblasts from patients with idiopathic pulmonary fibrosis exhibit genome-wide differences in DNA methylation compared to fibroblasts from nonfibrotic lung. PLoS One.

[248] Liu X, Sun SQ, Ostrom RS (2005). Fibrotic lung fibroblasts show blunted inhibition by cAMP due to deficient cAMP response element-binding protein phosphorylation. J Pharmacol Exp Ther.

